# Thalamic neuron models encode stimulus information by burst-size modulation

**DOI:** 10.3389/fncom.2015.00113

**Published:** 2015-09-23

**Authors:** Daniel H. Elijah, Inés Samengo, Marcelo A. Montemurro

**Affiliations:** ^1^Faculty of Life Sciences, The University of ManchesterManchester, UK; ^2^Statistical and Interdisciplinary Physics Group, Instituto Balseiro and Centro Atómico BarilocheSan Carlos de Bariloche, Argentina

**Keywords:** burst, information theory, multivariate analysis, neural code, reverse correlation, single neuron model, spike-triggered average, thalamus

## Abstract

Thalamic neurons have been long assumed to fire in tonic mode during perceptive states, and in burst mode during sleep and unconsciousness. However, recent evidence suggests that bursts may also be relevant in the encoding of sensory information. Here, we explore the neural code of such thalamic bursts. In order to assess whether the burst code is generic or whether it depends on the detailed properties of each bursting neuron, we analyzed two neuron models incorporating different levels of biological detail. One of the models contained no information of the biophysical processes entailed in spike generation, and described neuron activity at a phenomenological level. The second model represented the evolution of the individual ionic conductances involved in spiking and bursting, and required a large number of parameters. We analyzed the models' input selectivity using reverse correlation methods and information theory. We found that *n*-spike bursts from both models transmit information by modulating their spike count in response to changes to instantaneous input features, such as slope, phase, amplitude, etc. The stimulus feature that is most efficiently encoded by bursts, however, need not coincide with one of such classical features. We therefore searched for the optimal feature among all those that could be expressed as a linear transformation of the time-dependent input current. We found that bursting neurons transmitted 6 times more information about such more general features. The relevant events in the stimulus were located in a time window spanning ~100 ms before and ~20 ms after burst onset. Most importantly, the neural code employed by the simple and the biologically realistic models was largely the same, implying that the simple thalamic neuron model contains the essential ingredients that account for the computational properties of the thalamic burst code. Thus, our results suggest the *n*-spike burst code is a general property of thalamic neurons.

## 1. Introduction

Thalamic neurons can respond to input in either tonic or bursting modes (Steriade and Llinas, 1988; Sherman, 2001). Tonic firing typically consists of sequences of spikes whose temporal frequency can be modulated by the external stimulus, or by internal regulating mechanisms. Bursting involves highly correlated spikes fired in brief high-frequency packets underpinned by fairly rigid dynamic mechanisms (Huguenard and McCormick, 1992; McCormick and Huguenard, 1992; Guido and Weyand, 1995; Izhikevich, 2000, 2007). These two firing modes have been long thought to relate to distinct physiological functions. Tonic firing has often been related to awake and perceptive states where thalamic cells convey peripheral and cortical information to the cortex and other areas of the thalamus (Sherman, 1996). Bursting has been linked to sleep states, and originally, the rhythmic activity was assumed to isolate the thalamus from sensory input (Baker, 1971; McCormick and Feeser, 1990; McCormick and Pape, 1990; Jeanmonod et al., 1996). However, more recent experiments have provided evidence that thalamic bursts can also occur during awake states (Guido et al., 1992; Ohara et al., 2007; Marlinski and Beloozerova, 2014) and that they may convey information about external stimuli (Sherman, 1996; Reinagel et al., 1999; Lesica and Stanley, 2004).

Studies in the visual thalamus found that bursts mark the temporal or spatial position of salient or relevant visual stimuli (Cattaneo et al., 1981; Guido and Weyand, 1995; Livingstone et al., 1996; Alitto and Usrey, 2005; Alitto et al., 2005; Akerberg and Chacron, 2011). However, thalamic bursts may also provide a graded representation of the stimulus, based on reliably changing their internal structure to encode stimulus features. This internal structure, such as burst spike count (*n*) or duration, may provide downstream cells with information not conveyed by the temporal placement of bursts (Eyherabide and Samengo, 2010). For example, bursts recorded from the electro-sensory organ of weakly electric fish encoded the intensity of stimulus upstrokes through their inter-spike-interval (Oswald et al., 2007). In cat auditory cortical neurons, bursts with increasing spike counts became more narrowly tuned to the stimulus (Eggermont and Smith, 1996). Other auditory neurons in grasshoppers fire *n*-spike bursts that represent different stimulus features (Eyherabide et al., 2008; Creutzig et al., 2009). In addition, cricket auditory neurons fire *n*-spike bursts that signal the intensity of a bat echolocation ultrasound pulse, where larger bursts generate stronger avoidance behavior (Marsat and Pollack, 2010, 2012). In a cortical neuron model this *n*-spike burst code also represented different slopes and phases of driving stimuli (Kepecs et al., 2002; Samengo and Montemurro, 2010). Finally, Samengo et al. (2013b) found that neuron models possessing different dynamical mechanisms could encode different stimuli using *n*-spike bursts.

Still, a detailed characterization of a thalamic burst remains missing. Thalamic bursts are caused by slow sub-threshold depolarizations generated by a transient calcium *I*_*T*_ current, which in turn, is triggered by periods of quiescence or hyperpolarization (Rose and Hindmarsh, 1985; Huguenard and McCormick, 1992; McCormick and Huguenard, 1992). The strength of the Ca^+2^ conductance may therefore co-vary with different stimulus features (Bessaïh et al., 2008). Indeed, modeled and *in-vivo* thalamic responses display stimulus-mediated changes in both their calcium conductance and consequent burst spike count when driven with simple sinusoidal stimuli (Wang, 1994; Smith et al., 2000). The number of intra-burst spikes therefore encodes aspects of the stimulus. The first goal of this study is to determine the stimulus features encoded by burst duration.

Many thalamic neurons fire bursts underpinned by similar *I*_*T*_-current mechanisms (Jahnsen and Llinás, 1984; Steriade and Llinas, 1988; Sherman, 2001; Sherman et al., 2006; Wei et al., 2011). The second goal of this paper is to determine whether all *I*_*T*_-mediated bursting neurons share the same neural code, or whether the code depends on specific properties of individual neurons. To this end, we stimulated two thalamic neuron models with stochastic input (hereafter referred to as *the stimulus*). The first model contains a simple integrate-and-fire mechanism with an added slow *I*_*T*_ current to enable bursting (Smith et al., 2000). The second model is based on a more biologically realistic Hodgkin-Huxely model (Hodgkin and Katz, 1949; Fitzhugh, 1961; Krinskii and Kokoz, 1973; Rinzel, 1985; Rose and Hindmarsh, 1989) with two additional conductances (Wang, 1994). We investigated the neural code of these models using methods from information theory and reverse correlation. As shown below, we found that both models transmit information through largely the same neural code. Therefore, we propose that the basic computational properties of thalamic bursting neurons are determined by the dynamics of the *I*_*T*_ current and not by other biological details of these neurons.

## 2. Materials and methods

### 2.1. Thalamic neuron models

**Figure 1:**
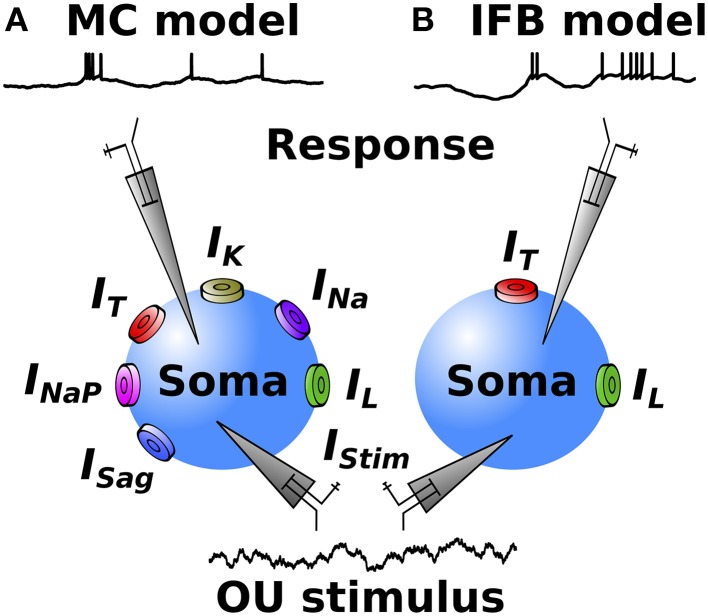
**Diagrams of the MC (A) and IFB (B) thalamic models**. The net synaptic input is represented by an Ornstein-Uhlenbeck (OU) process (bottom), here termed *the stimulus*. The models have no spatial structure. The conductances governing the evolution of the membrane potential (top traces) are marked.

To simulate thalamic neuron responses we employ two single compartment models: the multi-conductance (MC) and the integrate and fire or burst (IFB) models. The two models contain different levels of biological detail. Figure 1 shows a diagram of the MC (A) and the IFB (B) models, each driven by an Ornstein-Uhlenbeck (OU) stimulus, and producing membrane voltage responses.

The IFB model is based on integrate and fire equations and is the simplest model used here. It contains an additional voltage-gated calcium (*I*_*T*_) conductance that permits bursting. Unless stated explicitly, we used the original parameters defined by Smith et al. (2000) where the membrane potential is governed by

(1) $$C_{m}\text{d}V/\text{d}t\;=I_{Stim}-(I_{T}+I_{L}).$$

The leak conductance *I*_*L*_ was defined as *I*_*L*_ = *g*_*L*_(*V*_*m*_ − *E*_*L*_) where *g*_*L*_ and *E*_*L*_ are the conductance and reversal potential, respectively. The *I*_*T*_ current is defined as *I*_*T*_ = *g*_*T*_*m*_∞_*h*(*V*_*m*_ − *E*_*T*_) where the activation function *m*_∞_(*V*_*m*_) = Θ(*V*_*m*_ − *V*_*h*_) is modeled in terms of a Heaviside step function Θ(*x*) = 1 if *x* > 0, and Θ(*x*) = 0 if *x* ≤ 0. The inactivation variable *h* is governed by d*h*∕d*t* = −*h*∕τ^−^_*h*_ when *V*_*m*_>*V*_*h*_, and by d*h*∕d*t* = (1−*h*)∕τ^+^_*h*_ otherwise. The channel is hence open for *V*_*m*_ < *V*_*h*_, and the inward Ca^2+^ current depolarizes the neuron. However, as *V*_*m*_ surpasses *V*_*h*_, the inactivating variable *h* drops to zero with a time constant τ^−^_*h*_ = 20 ms, thereby closing the channel. When the membrane potential decreases below *V*_*h*_, *h* returns to unity with a slow time constant τ^+^_*h*_ = 20 ms, re-opening the channel. We use the same parameters as Smith et al. (2000), namely, *C_m_* = 2 μFcm^−2^, *gL* = 0.035 mScm^−2^, *E*_*L*_ = −65 mV, *g*_*T*_ = 0.07 mS, *E*_*T*_ = 120 mV. Spiking and bursting are controlled by two thresholds *V*_θ_ = −35 mV and *V*_*h*_ = −60 mV, respectively. If *V*_*m*_ > *V*_θ_ then a spike is fired, and *V*_*m*_ is reset to the value *V*_reset_ = −50 mV. Finally, we generate a tonic firing variant of the IFB model by setting *g*_*T*_ = 0 mS to remove bursting. The modified model is termed *the tonic IFB model* (IFB-T).

The more complex MC model was built using Hodgkin-Huxely conductances with further conductances simulating burst behavior (Hodgkin and Huxley, 1952; Wang, 1994). We ran the model using equations and parameters employed by Wang (1994). The membrane voltage is governed by

(2) $$C_{m}\text{d}V_{m}/\text{d}t\;=I_{Stim}-(I_{L}+I_{Na}+I_{K}+I_{T}+I_{Sag}+I_{NaP}).$$

*I*_*L*_ is defined as *I*_*L*_ = *g*_*L*_(*V*_*m*_−*E*_*L*_), where *g*_*L*_ = 0.1 mS and *E*_*L*_ = −72 mV. The *I*_*Na*_ current is defined as *I_Na_* = *g*_*Na*_*m^3^*_∞_ (0.85−n)(*V_m_* − *E_Na_*), where *g*_*Na*_ = 42 mS and *E*_*Na*_ = 55 mV. The activation function *m*_∞_ follows the general equilibrium equation *x*_∞_ = α_*x*_∕(α_*x*_+β_*x*_), where *x* may be *m, n* or *h*. The gating variables α_*m*_ and β_*m*_ are defined as α_*m*_(*V*_*m*_) = 0.1(*V*_*m*_+29.7−σ_*Na*_)∕(1−exp(−(*V*_*m*_+29.7−σ_*Na*_)∕10)) and β_*m*_(*V*_*m*_) = 4(exp(−0.0556(*V*_*m*_+54.7−σ_*Na*_))), with σ_*Na*_ = 3. The variable *n* participates both in the equation for *I*_*Na*_ and *I*_*K*_ (see below). In the equation for *I*_*Na*_, it is preceded by a negative sign. Therefore, the term 0.85−*h* operates as an inactivating variable. Its evolution is governed by d*n*∕d*t* = 200(*n*_∞_−*n*)∕7(τ_*n*_). Here, *n*_∞_ follows the general equilibrium expression, with α_*n*_(*V*_*m*_) = 0.01(*V*_*m*_+45.7−σ_*K*_)∕(1−exp(−0.1(*V*_*m*_+45.7−σ_*K*_))), β_*n*_(*V*_*m*_) = 0.125(exp(−(*V*_*m*_+55.7−σ_*K*_)∕80)), τ_*n*_(*V*_*m*_) = 1∕(α_*n*_+β_*n*_) and σ_*K*_ = 10. The *I*_*K*_ current is defined as *I_K_* = *gKn^4^*(*V_m_* − *E_K_* where *g*_*K*_ = 30 mS and *E*_*K*_ = −80 mV. The variable *n* appears here with a positive sign, and therefore acts as an activation variable. The *I*_*T*_ current is defined as *I*_*T*_ − *g*_*T*_S^3^_∞_*h*(*V*_*m*_ − *E*_*T*_) where the activation variable *S*_∞_ is *S*_∞_ = 1∕(exp(−(*V*_*m*_+65)∕7.8)) and the inactivation variable *h* is governed by d*h*∕d*t* = 2(*h*_∞_−*h*)∕τ_*h*_, with *h*_∞_(*V*_*m*_) = 1∕(1+exp(−(*V*_*m*_−θ_*h*_)∕*k*_*h*_)) and τ_*h*_ = *h*_∞_exp((*V*_*m*_+162.3)∕(17.8)+20). The parameters are *g*_*T*_ = 0.3 mS, *E*_*T*_ = 120 mV, θ_*h*_ = −81 mV and *K_h_* = 6.25 mV^−1^. The *I*_**Sag**_ current is defined as *I*_*Sag*_ = *g*_*Sag*_*H*^2^(*V_m_* − *E*_*sag*_). Its gating variable *H* is governed by d*H*∕d*t* = (*H*_∞_−*H*)∕τ_*H*_ where the equilibrium variable is *H*_∞_ = 1∕(1+exp((*V*_*m*_+69)∕7.1)) and τ_*H*_ = 1000∕(exp((*V*_*m*_+66.4)∕9.3)+exp(−(*V*_*m*_+81.6)∕13)). The persistent sodium current *I*_*NaP*_ is defined as *I*_*NaP*_ − *gNaP*^m^3^^_*P∞*_(*V_m_* − *E_Na_*), with *m*_*P∞*_, α_*Pm*_ and β_*Pm*_ follow the general expressions for *m*_∞_. The tuning constant σ_*NaP*_ = −5 and its conductance is *g*_*NaP*_ = 9 mS. Like the IFB-T model, we also produced a tonic variant (MC-T) by removing all burst-associated conductances (*g*_*T*_ = *g*_*Sag*_ = *g*_*NaP*_ = 0 mS) and increasing *g*_*Na*_ to 120 mS. For both tonic and bursting models, the membrane capacitance is 1 μFcm^−2^

### 2.2. Driving stimulus

We stimulated the models with an Ornstein-Uhlenbeck (OU) colored noise signal (Uhlenbeck and Ornstein, 1930; Smith, 1992; Gillespie, 1996; Rauch et al., 2003; Bibbona et al., 2008). The OU process is governed by the equation d**OU**∕d*t* = ((μ_*OU*_−**OU**)+*uξ*)∕τ_*OU*_ where μ_*OU*_ is the stimulus' mean, τ_*OU*_ is the correlation time, ξ is Gaussian distributed white noise with unit variance (Arnold, 1974) and *u* is an amplitude scaling factor defined as $u=\sigma_{OU}\sqrt{2(\tau_{OU})}$. Two constraints restrict the allowed fluctuations. First, the process contains a characteristic autocorrelation time constant τ_*OU*_, and thus cannot vary at infinitely small timescales. Second, the process tends to take values not far from its mean over extended periods of time. Importantly, the decay of spectral power with increasing frequency matches the power spectra of membrane fluctuations (Verveen and Derksen, 1965; Derksen and Verveen, 1966; Verveen and Derksen, 1968; Verveen and DeFelice, 1974).

To analyze the effects of spike correlations on both the reliability and the information content of the response, we gather neuron responses to multiple repetitions, or trials, of a stimulus. We drove the models with 100 trials of repeated 15 s OU stimuli. For each trial, the OU stimulus was “corrupted” with a white noise component that varied from trial to trial. The strength of added noise was defined using a Noise:Signal (*N*:*S*) ratio where *N*:*S* = 5. The amplitude of the OU stimulus for each model were 0.8μA (MC), 0.5μA (IFB), 5μA (MC-T), and 3μA (IFB-T). These values produced physiologically plausible firing rates within the range of 5.8 ± 3.6 Hz and cross-trial variability of 0.43 ± 0.05 ms (Montemurro et al., 2007a). When analyzing the stimulus selectivity of thalamic *n*-spike bursts no noise component is required, we therefore set the stimulus parameters as follows: σ_*OU*_ = 1μ*A*, μ_*OU*_ = 0μ*A*, and τ_*OU*_ = 5 ms. Simulations were also run for τ_*OU*_ equal to 2.5 and 10 ms to test the robustness of the results.

### 2.3. Numerical integration

Both models were integrated with a backward-implicit implementation of the Euler-Murayama stochastic integration method (Kloeden and Platen, 1999), with a time step *h* = 0.02ms (Iserles, 2009). For all practical matters, identical responses were obtained with smaller (*h*∕2) or larger (2*h*) time steps.

### 2.4. Sorting *n*-spike bursts

To separate different response events, the autocorrelation function of neural responses was calculated. Spikes fired within bursts produced peaks at timescales between 2 and 10 ms. A burst was defined as a group of spikes separated by less than 10 ms. Results were also verified using a 6 ms intra-burst ISI threshold. For every burst, its spike count *n*, onset time *t*_*i*_ and duration *D* were collected. Single spikes were defined here as 1-spike bursts, the largest burst collected here contained 6 spikes.

### 2.5. Spike train variance analysis

When presented with multiple trials of the same stimulus, neurons do not produce identical responses. In particular, the number of spikes in a given time window may fluctuate. The Fano factor is a relative measure of the variance of the spike count *V*(*t*) with respect to the mean *M*(*t*) (Berry and Meister, 1998; Scaglione et al., 2011; Quian Quiroga and Panzeri, 2013). To estimate such means and variances, the two models were driven by 100 trials of noise-corrupted stimuli. Their responses were discretized into 20 ms non-overlapping windows, and the spike count in each window was computed. The values of *M*(*t*) and *V*(*t*) were estimated as the mean and the variance across trials.

### 2.6. Estimating the amount of information carried by the response

A major goal of this study was to find whether bursts constitute an informative neural code. We addressed this problem by applying information theory, which provides a model-independent framework to quantify the amount of information about the stimulus that can be read from the neural response (Shannon, 1948; Borst and Theunissen, 1999; Cover and Thomas, 2006). Specifically, we calculated Shannon mutual information *I* to quantify the information carried by the entire response. We also used a derived information measure Δ*I* (Panzeri et al., 1999; Panzeri and Schultz, 2001; Nirenberg and Latham, 2003; Pola et al., 2003; Latham and Nirenberg, 2005) to account for the stimulus-modulated correlations. To estimate Shannon mutual information, the two models were driven with many repetitions of one time-dependent stimulus. In each repetition, the input signal was corrupted with additive noise, that varied from trial to trial. Spike counts were taken in non-overlapping time windows of duration *T*. The position of spikes within each window was measured with a precision Δ*t* = 5 ms so that each response window was composed of *L* = *T*∕Δ*t* bins. The result is a collection of response words *r* = [*r*_1_, *r*_2_, *r*_3_…*r*_*L*_], where *r*_*i*_ represents the spike count within the *i*th bin (Strong et al., 1998; Montemurro et al., 2007a).

The conditional probability of observing a particular response word *r* at a given time *t*, is given by *P*(*r* ∣ *t*). Under the ergodicity assumption (Strong et al., 1998), different times *t* tag different stimulus histories. The probability of observing a response irrespective of time (i.e., of stimulus history) is therefore *P*(*r*) = 〈 *P*(*r*∣*t*)〉_*t*_, where 〈 …〉_*t*_ represents a temporal average.

We then calculated Shannon mutual information between the response and stimulus (Shannon, 1948; de Ruyter van Steveninck and Bialek, 1988; Strong et al., 1998)

(3) $$I\;=\;{\langle \sum_{r}P(r|t)\log_{2}\left[\frac{P(r|t)}{P(r)}\right]\rangle }_{t}.$$

The information rate is defined as the limit for *T* → ∞ of the ratio *I*∕*T*. In practice, we found that this ratio tends to a well-defined limit for *T*>20 ms. As *T* is increased further, a stable value is obtained inside the range *T* ∈ [20, 60] ms. For *T* > 60 ms, however, the estimates deteriorate rapidly due to limited sampling. For these reasons, here we set *T* = 40 ms (*L* = 8) bins to avoid sampling limitations. For shortness, information rates are reported just as *information* throughout the paper.

The information *I* contains the contributions of independent and correlated spiking activity. Previous studies have reported information being carried by trains of independent spikes. For example, different stimuli may induce different spike counts within a set time window (Henry et al., 1973; Darian-Smith et al., 1979). Alternatively, neurons may use sequences of precisely-timed spikes to encode stimuli (Thorpe, 1990; de Ruyter van Steveninck et al., 1997; Petersen et al., 2002; VanRullen et al., 2005; Montemurro et al., 2007a). In both cases, stimuli modulate the firing of statistically independent spikes while spike correlations (patterns of spikes) convey little or no stimulus information. However, spike correlations may become informative if they reliably alter their structure to different stimuli. Since bursts induce strong spike correlations and contain complex, possibly stimulus-dependent internal structure, they could underlie spike correlation coding in thalamic neurons.

We assessed the amount of information carried by stimulus-modulated correlations by using the measure Δ*I* (Panzeri et al., 1999; Panzeri and Schultz, 2001; Nirenberg and Latham, 2003; Latham and Nirenberg, 2005; Montemurro et al., 2007a), also called *I*_*corr*−*dep*_ (Pola et al., 2003). This measure is zero when stimulus-modulated correlations encode no information in the response (Pola et al., 2003). Starting from the conditional distribution of the response *P*(*r*∣*t*) it is possible to compute the independent distribution *P*_*ind*_(*r*∣*t*), where all correlations across time are ignored (Nirenberg and Latham, 2003; Montemurro et al., 2007b). Then, defining *P*_*ind*_ as *P*_*ind*_(*r*) = 〈 *P*_*ind*_(*r*∣*t*)〉_*t*_, the quantity Δ*I* is given by

(4) $$\Delta I\;=\;{\langle \sum_{r}P(r|t)\log_{2}\left[\frac{P(r|t)P_{ind}(r)}{P_{ind}(r|t)P(r)}\right]\rangle }_{{}_{{}_{t}}}.$$

All information estimations are affected by an upward bias due to finite sampling which, if left uncorrected, lead to an overestimation of the actual information (Panzeri and Treves, 1996; Panzeri et al., 2007). To account for the bias, we employed a shuffling correction procedure for both *I* and Δ*I* estimates (Montemurro et al., 2007b; Panzeri et al., 2007).

### 2.7. Defining instantaneous stimulus features

Bursts have been shown to be capable of signaling basic stimulus quantities, such as phase (Samengo and Montemurro, 2010), slope (Kepecs et al., 2002), and transitions between hyperpolarizing and depolarizing stimuli (Alitto et al., 2005). For each event-triggered stimulus window, we analyzed six stimulus features that either alter the strength of burst currents (Rose and Hindmarsh, 1985; McCormick and Feeser, 1990; Huguenard and McCormick, 1992; Huguenard, 1996) or have been previously shown to affect burst size (Kepecs et al., 2002; Alitto et al., 2005; Samengo and Montemurro, 2010). Defining *t* as the time relative to the first burst spike, which occurs at *t* = 0, these features are

1. The stimulus amplitude at burst onset *x*(*t* = 0).
2. The stimulus minimum in a time window preceding burst onset min[*x*(*t*)], where *t* is taken from a window spanning the stimulus prior to burst onset, starting at *t*+*T*_**start**_—with *T*_**start**_ < 0—and ending at burst onset *t* = 0.
3. The stimulus instantaneous slope at burst onset, given by d*x*∕d*t*|_*t* = 0_.
4. The amount of negative (hyperpolarizing) charge entering the neuron prior to burst onset $N_{int}=\int_{T_{start}}^{0}x(t)\Theta [-x(t)]\text{d}t$.
5. The amount of positive (depolarizing) charge entering the neuron after burst onset, calculated as $P_{int}=\int_{0}^{T_{end}}x(t)\Theta [x(t)]\text{d}t$.
6. The stimulus phase at burst onset, calculated as the phase angle between the real and imaginary components of the Hilbert transformed stimulus, *x*(Φ) = arctan{ImHil[*x*(*t*)]∕ReHil[*x*(*t*)]}, where Im and Re indicate the imaginary and real part, respectively (Hahn, 1996; Oppenheim and Schafer, 2010).

We refer to these stimulus features collectively as *F*.

### 2.8. The information encoded by bursts about instantaneous stimulus features

To quantify the extent up to which specific stimulus features (*F*) are encoded by *n*-spike bursts, we estimated Shannon mutual information between *F* and burst size (Samengo and Montemurro, 2010). For each *n*-spike burst initiated at time *t*_0_, we annotated the value of feature *F* at time *t*_0_+*t*. Information values were computed as a function of *t*, that is, of the temporal difference between burst onset an the stimulus feature. To calculate the probabilities needed for the computation of information, the values of *F* were discretized to give $\tilde{F}$, each descretized value of $\tilde{F}$ is termed *f*, where $f\in \tilde{F}$. The probability of *f* conditioned to a burst of a given size *n* is *P*(*f*∣*n*). The marginal distribution is *P*(*f*) = < *P*(*f*∣*n*)>_*n*_. The total entropy then reads,

(5) $$H(\tilde{F})\;=-\sum_{f\in \tilde{F}}P(f)\log_{2}P(f).$$

Similarly, the conditional entropy is

(6) $$H(\tilde{F}|N)\;=-\sum_{n\in N}P(n)\sum_{f\in \tilde{F}}P(f|n)\log_{2}P(f|n),$$

where *N* is the set of all possible burst sizes *n*. Shannon mutual information between burst size and the stimulus feature is $I(\tilde{F};N)=H(\tilde{F})-H(\tilde{F}|N)$.

The discretization of *F* was done by binning into *M* = 32 equally populated bins. Any finite value of *M* inevitably results in some information loss. If an infinite amount of data were available, the information estimate would approach the true value as *M* → ∞. However, if the size of the sample is finite, increasing *M* amplifies the effects of bias, especially when computing the conditional entropy $H(\tilde{F}|N)$ (Panzeri et al., 2007). We tested a range of discretization bins for *M* = 2^[2, 3, 4…12]^, using a minimum of 10^4^ samples, and found that *M* = 32 provided a precise estimate of the information with minimal bias (see Supplementary Figure 1). Any residual bias still present when *M* = 32 was removed by means of a bootstrap procedure. Specifically, information was estimated on surrogate data sets created from the original data, by randomly shuffling the values of the features corresponding to each burst size, thus destroying any statistical relationship between them. Due to the undersampling bias, the estimated information in the surrogate data set is typically still above zero. The obtained value is then subtracted from the mutual information estimated on the original data.

### 2.9. Event-triggered stimulus averages

While Shannon information quantifies the degree of correspondence between stimulus and response, it does not identify the stimulus features encoded by the response. We addressed this issue by reverse correlation techniques. A first order analysis involves calculating *n*-event-triggered averages (Samengo et al., 2013a). If the first spike of an *n*-spike burst was fired at time *t*, we extracted the stimulus surrounding the burst using a window located at [*t*+*T*_**start**_, *t*+*T*_**end**_]. For the IFB model *T*_**start**_ = −500ms and *T*_**end**_ = 100ms, with a bin size of Δ*t* = 2ms. For the MC model, *T*_**start**_ = −250ms, *T*_**end**_ = 50ms, and Δ*t* = 1ms. For each *n*, we stacked stimulus windows together to form a matrix of all *n*-event triggering stimuli *x*_*n*_. Event-triggering stimuli may contain only a small set of stimulus fluctuations that modulate the neuron response (Agüera y Arcas and Fairhall, 2003; Schwartz et al., 2006). A simple way to isolate this subset of stimuli is to calculate the *n*-event triggered average (*n*-ETA), which represents the average stimulus deflection surrounding an *n*-spike event de Boer and Kuyper (1968); Thompson and Radpour (1991); Agüera y Arcas and Fairhall (2003); Schwartz et al. (2006).

By comparing *n*-ETAs associated with different *n*, we may identify changes in stimulus preference. Those changes are candidate features that may be discriminated by downstream neurons reading *n*-spike bursts.

### 2.10. Event-triggered covariance analysis

Occasionally, bursts are triggered by more than a single stimulus feature. To detect multiple relevant features, reverse correlation methods can be extended to the second order, resulting in event-triggered covariance analysis (ETC) (Chichilnisky, 2001; Agüera y Arcas and Fairhall, 2003; Rust et al., 2004; Schwartz et al., 2006; Samengo and Gollisch, 2013). In brief, ETC analysis finds a set of axes in a *k*-dimensional space where the variance of *n*-triggering stimuli differs significantly from that of randomly selected stimuli (de Ruyter van Steveninck and Bialek, 1988; Brenner et al., 2000; Rust et al., 2005; Fairhall et al., 2006; Pillow and Simoncelli, 2006; Schwartz et al., 2006; Samengo and Gollisch, 2013). In order to eliminate the bias introduced by the correlations in the prior stimulus distribution, we calculate the “relative covariance matrix” through a matrix multiplication between the inverse covariance matrix of randomly selected stimuli and the *n*-triggered stimuli covariance matrix (Samengo and Gollisch, 2013; Samengo et al., 2013b). Eigenvalues of the relative covariance matrix that significantly deviate from unity are associated with eigenvectors whose directions in the *k*-dimensional stimulus space indicate the features evoking *n*-spike triggering bursts.

### 2.11. Multi-discriminant analysis

Reverse correlation techniques reveal the stimulus dimensions that are relevant in shaping the distribution of stimuli triggering *n*-spike bursts. Relevance is assessed by comparing the mean or the variance of such stimuli to the mean or variance of a random set of stimuli. The features described by the relevant eigenvectors obtained for a specific value of *n*, hence, are associated with the presence (or absence) of burst containing *n* spikes. Those features may or may not be the same, for different *n*-values. Therefore, although they are useful to predict whether a burst of specifically *n* spikes is expected, they are not necessarily useful to discriminate between different *n*-values. In order to identify the stimulus features that optimally discriminate between bursts of different duration, we turn to multi-discriminant analysis (MDA) (Duda et al., 2001; Kepecs and Lisman, 2003). The criterion to define the optimal discrimination is based on Fisher's Linear Discriminant (Fisher, 1936).

The method considers *n*-burst triggering stimuli as vectors in a *k*-dimensional space, where *k* is the number of bins in the window used to represent the time-dependent stimulus associated to each *n*-spike burst. Stimuli evoking bursts of a specific size *n* typically share some common features, and therefore tend to form a cluster in a given region of the *k*-dimensional space. The goal is to find the stimulus dimension where the projections of the stimulus vectors conforming the clusters corresponding to different *n*-values are optimally discriminated. The method not only provides the best dimension, but also the second best, third best, and so on. The solution is given as an ordered set of *k* orthogonal axes **V**_*j*_ (*j* = 1…*k*), in decreasing order of effectiveness in discriminating clusters. The components of each vector represent the weights with which each time bin participates in the selected axis (Duda et al., 2001; Kepecs and Lisman, 2003).

Once the vectors are obtained, in order to calculate the mutual information corresponding to the vector **V**_*j*_, we project every each stimulus window triggering an *n*-burst onto the (normalized) **V**_*j*_, obtaining a set of scalar quantities *y*_*ij*_, where the index *i* runs over the set of stimulus windows. Using the descretized projected values (*M* = 32) we calculated the distributions *P*(*y*_*j*_∣*n*) and *P*(*y*_*j*_), and the associated entropies *H*(*Y*_*j*_) and *H*(*Y*_*j*_∣*N*) using Equations (5, 6). The mutual information was then found by computing *I*(*Y*_*j*_; *N*) = *H*(*Y*_*j*_)−*H*(*Y*_*j*_∣*N*)). We limited the analysis to the most significant discriminants, which in our data included *j* = 1 and *j* = 2.

## 3. Results

### 3.1. Spike train variability in the presence of bursts

**Figure 2:**
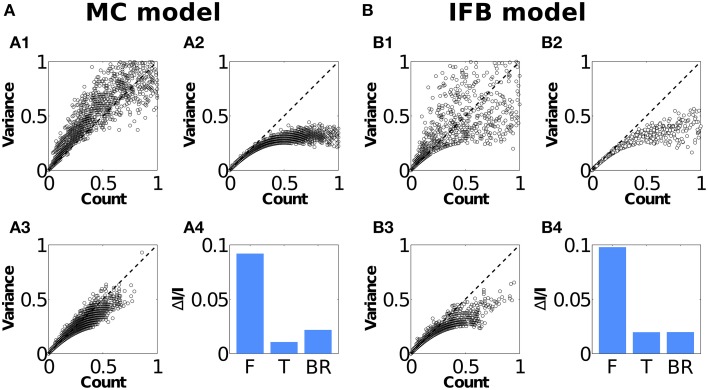
**Analysis of spike-train correlations**. Mean and variance of the spike count in a 20 ms window from 100 trials, for the full responses **(A1, B1)**, tonic model responses **(A2, B2)**, and full responses with bursts removed **(A3, B3)**. The fraction of information contained in stimulus-modulated correlations (Δ*I*∕*I*) is shown for MC **(A4)** and IFB **(B4)** models, for the full response (F), tonic response (T), and the full response with bursts removed (BR). Response window size *L* = 40 ms.

The variability of neuron responses to repeated presentations of a stimulus is usually quantified by means of the Fano factor, that is, by the ratio between the spike count variance and the spike count mean, both measured in a given time window. The Fano factor of a neuron governed by a Poisson firing mechanism is equal to one. Systematic deviations from unity indicate the presence of correlations between spikes (Berry and Meister, 1998; Panzeri et al., 1999; Quian Quiroga and Panzeri, 2013). To quantify how bursts affect the variability of spike responses in the neuron models, we computed the mean *M*(*t*) and variance *V*(*t*) of the spike count in 20 ms non-overlapping windows across 100 trials (see Section Materials and Methods), as shown in Figures 2A1–A3,B1–B3. Both models contained windows with sub-Poissonian (*V*(*t*)∕*M*(*t*) < 1) and with super-Poissonian (*V*(*t*)∕*M*(*t*)>1) behavior, see Figures 2A1,B1. The points below the diagonal are a direct consequence of negative correlations induced by the refractory period (Berry and Meister, 1998; Montemurro et al., 2007a). To test whether the points above the diagonal were a consequence of bursting, we ran a modified version of the models (termed the IFB-T and MC-T models) that were incapable of producing intrinsic bursting (see Section Materials and Methods). As shown in Figures 2A2,B2, their responses produced no super-Poissonian data. Additionally, we also tested the effect of removing all bursts from the responses of the original (bursting) models and found that super-Poissonian regions were drastically reduced, as seen in Figures 2A3,B3. Both tests indicate that super-Poissonian responses (*V*(*t*)∕*M*(*t*)>1) are indeed due to bursting rather than other causes like, for instance, fluctuations due to insufficient sampling.

One important question is whether bursts can actually encode information about the stimulus. To address this issue, we computed the Shannon mutual information *I* between the stimulus and the response. We then estimated how much of this information was due to stimulus-modulated correlations using the measure Δ*I* (Panzeri et al., 1999; Panzeri and Schultz, 2001; Nirenberg and Latham, 2003; Pola et al., 2003; Montemurro et al., 2007a) (see Section Materials and Methods). Stimulus-modulated correlations may not only be due to bursting. In order to uniquely isolate the contribution of bursts to coding, we computed *I* and Δ*I* for both the MC and IFB models, for their non-bursting counterparts, and for the spike trains generated by the original models with bursts removed.

We estimated the total information rates for the MC and IFB models as *I* = 15.2±0.7 bits/s and 13.8±0.5 bits/s, respectively, while the MC-T and IFB-T models transmitted 14.0±0.6 bits/s and 12.8±0.6 bits/s, respectively. In Figures 2A4,B4, the values of Δ*I* amounted to 9.3% (MC) and 9.8% (IFB) of the total information *I*. The tonic firing MC-T and IFB-T models conveyed much less information in correlations, 1.1 and 2.0%, respectively. Responses where bursts were removed directly (BR) also conveyed significantly less information through stimulus-modulated correlations, yielding 2.3% (MC) and 1.1% (IFB). Hence, the spike correlations induced by bursting convey information about the stimulus, which is consistent with previous *in-vivo* results (Montemurro et al., 2007a).

### 3.2. Reverse correlation analysis

**Figure 3:**
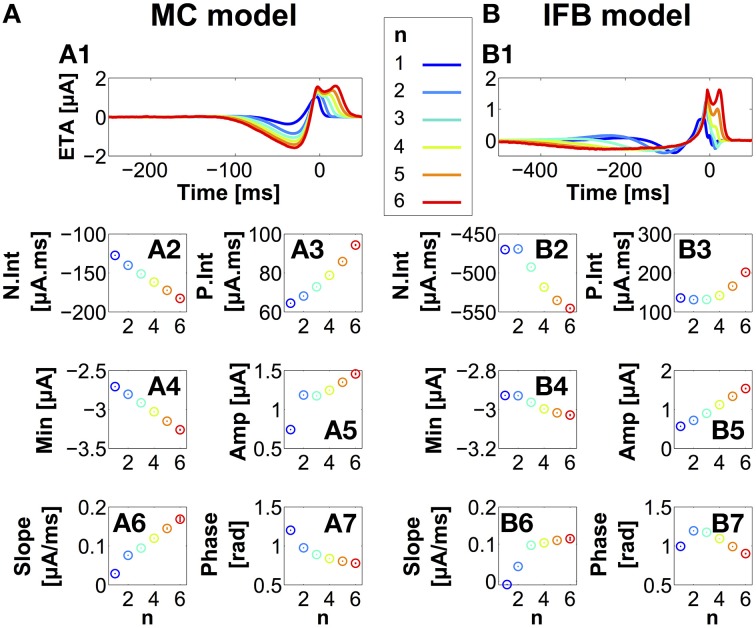
**Stimulus features associated with bursts containing ***n*** spikes**. Event-triggered averages for the MC **(A1)** and IFB **(B1)** models, for different *n*-values (see color key at top center). Time = 0 ms marks the first spike in the burst. The values of several stimulus features are averaged and plotted as a function of *n* **(A2–A7, B2–B7)**, including pre-onset hyperpolarizing stimulus charge (N.int) (2), post-onset depolarizing stimulus charge (P.int) (3), stimulus minimum prior to onset (4), stimulus amplitude (5), slope (6), and stimulus phase (7). Amplitude, slope and phase are all calculated at burst onset. Error bars represent ±1SE of the mean. Mean phase and the corresponding error bars are calculated with circular statistics.

To determine the stimulus features encoded by bursts of different length, we used reverse correlation methods (Rieke, 1997; Chichilnisky, 2001; Samengo et al., 2013b). We stimulated both thalamic models with an OU stimulus current, and we identified the events ranging from single spikes to 6-spike bursts. Sections of the stimulus surrounding each of these events (event-triggered stimuli) were then collected. In all cases, the number of event-triggered stimuli for each *n*-spike event exceeded 1 × 10^4^. Figures 3A1,B1 shows *n*-spike event-triggered stimulus averages (*n*-ETAs) for different burst sizes from the MC (A1) and IFB (B1) models (see Section Materials and Methods). For both models, all *n*-ETAs contain a pre-onset hyperpolarization followed by a post-onset depolarization (Time = 0 ms indicates burst onset). The double-peak structure observed for positive times in both models closely resembles the results obtained by Samengo et al. (2013b) for quadratic bursters. The amplitude of the pre and the post-onset features grows with increasing *n*. While both the MC and IFB bursts have qualitatively similar stimulus preference, the hyperpolarizing trajectory of the stimulus prior to burst onset is longer in the IFB model. Longer IFB bursts are evoked by hyperpolarizations of increased duration, whereas longer MC bursts are evoked by hyperpolarization of increased (negative) amplitude.

In other brain areas, bursts were shown to encode instantaneous stimulus parameters, like slope (Kepecs et al., 2002) or phase (Samengo and Montemurro, 2010). In order to identify the stimulus features encoded by thalamic bursts, we explored the relation between burst size and several candidate stimulus properties. The results are shown in Figures 3A2–A7,B2–B7. We tested the following stimulus features: the negative (A2, B2) and positive (A3, B3) stimulus charge entering the neuron prior to burst onset, denoted, respectively as N.int and P.int; the stimulus minimum prior to burst onset (A4, B4); the stimulus amplitude (A5, B5), slope (A6, B6), and phase (A7, B7), the latter three computed at burst onset. In all cases, the values were averaged and plotted as a function of burst size *n*.

Almost all parameters varied monotonically with increasing *n*, implying that to a greater or lesser extent, all the tested stimulus features were encoded in the number of spikes per burst. For the stimulus amplitude driving the MC model (A5) and negative stimulus integral driving the IFB model (B2), single-spike events (*n* = 1) slightly departed from the common trend. Interestingly, the case of *n* = 1 is special. While for the purposes of the analysis we take all *n* = 1 events as single spikes, the ensemble of all such events can be separated into two distinct classes. Some of the single spikes are fired without activation of the *I*_*T*_ current; these are purely tonic spikes. There are other events with *n* = 1, however, that are fired with a significant activation of the *I*_*T*_ current; in a strictly dynamical sense, these are 1-spike bursts. A more detailed discussion of this distinction is given as supplementary information.

In summary, both models encode similar ranges of instantaneous stimulus parameters with the same overall increasing or decreasing trends. The similarity survives despite the different levels of biological complexity contained in the models.

The stimulus features encoded by burst duration are not necessarily detected by arithmetic averages. In principle, bursts of a specific size could be triggered by several independent stimulus features that, when averaged together, may either cancel out to zero, or be combined into a new feature that by itself, does not trigger bursts. In order to detect the relevant independent features, we turn to a second-order version of reverse correlation analysis, and calculate the event-triggered covariances (see Section Materials and Methods). The idea is to search for stimulus directions associated with *n*-spike bursts that have increased or decreased variance, as compared to a random selection of stimuli. The features described by those directions are the uncorrelated components of the stimulus that are associated with an *n*-spike burst. The ETA is a specific linear combination of such features (Samengo and Gollisch, 2013).

**Figure 4:**
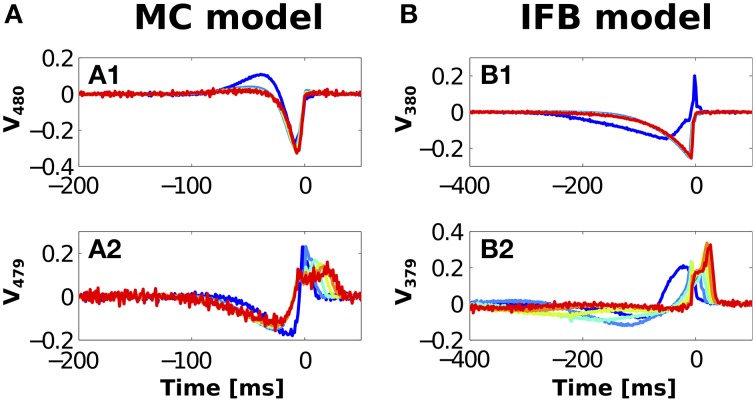
**Most relevant vectors obtained by covariance analysis, for the MC (A) and the IFB (B) models and different ***n***-values (see color code in Figure 3)**. Vectors with smallest variance [*V*_480_ for the MC **(A1)** and *V*_380_ for the IFB **(B1)**] and second smallest variance [*V*_479_ for the MC **(A2)** and *V*_379_ for the IFB model **(B2)**].

For the two models studied here, and for all values of *n*, the two most relevant directions corresponded to decreased variances. The corresponding eigenvectors are displayed in Figure 4. The subsequent axes are associated to eigenvalues that are closer to unity, and represent weak stimulus features with no clear *n*-dependent structure.

Notably, in both models the most relevant eigenvector only contained pre-onset structures. The second eigenvector, instead, contained both pre- and post-onset features. Hence, some of the features triggering bursts (the ones in the most relevant eigenvector) are statistically uncorrelated with the features terminating bursts (see Samengo et al., 2013b for similar examples in other types of bursting neurons).

Qualitatively, the lowest-variance STC vector (panels A1 and B1) represents the pre-onset priming of the *I*_*T*_ current in both models followed by a depolarizing “event trigger.” For the MC model (A1), 1-spike bursts (blue curve) require an initial membrane depolarization at −50 ms followed by the priming hyperpolarization/trigger. Single IFB model spikes are evoked by a hyperpolarization followed by a sharp depolarization at onset. In both models, larger events (*n*>1) display hyperpolarization triggers whose shape is largely unchanged for different *n*, indicating little discrimination.

The second lowest variance vector *V*_479_ evoking MC model responses (A2) displays *n*-dependent structure both pre- and post-onset. Pre-onset *n*-dependent regions range from −90 ms to onset, the majority of this dependence stems from the difference between vectors associated with single spikes and multi-spike bursts. Post-onset, these vectors show *n*-dependent structure extending up to 38 ms after burst onset.

For the IFB model, the lowest variance ETC vector *V*_380_ (B1) shows a difference in stimulus preference between single spikes and bursts beginning −200 ms before onset. The second lowest variance vectors *V*_379_ (B2) showed a graded dependence on *n*. As *n* increases, the pre-onset hyperpolarization decreases in depth but increases in duration.

The stimulus features that remain unchanged as *n* varies are encoded in the presence or absence of bursts, but not in the distinction between bursts of different lengths. Distinctions between bursts can only encode stimulus features that vary significantly with *n*. In order to make these statements quantitative, in the following sections we calculate the amount of information encoded by bursts.

### 3.3. Encoding instantaneous stimulus properties

**Figure 5:**
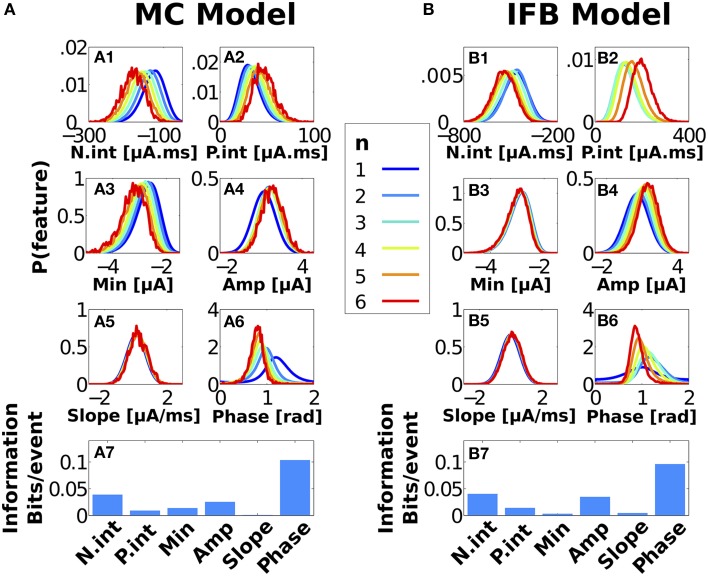
**Probability density functions of stimulus features triggering bursts of ***n*** spikes and associated information, for MC (A) and IFB (B) models**. Instantaneous stimulus features **(A1–A6, B1–B6)**, with the same color code as in Figure 3 (see key). For better visualization, distributions are plotted with a resolution of *M* = 256 bins. Information values (displayed in **A7, B7**) were calculated with a coarser binning *M* = 32 to reduce estimation bias.

In Figures 3A2–A7,B2–B7, we showed that burst size varied mostly monotonically with the mean value of several instantaneous stimulus features. However, an analysis of mean values does not suffice to assess the quality of the encoding: Variances and higher moments also matter. We therefore analyze the whole distribution of stimulus parameters eliciting bursts of a given size. In Figures 5A1–A6,B1–B6 we plot the normalized histograms of instantaneous stimulus features evoking bursts of different duration. Intuitively, if the distributions associated to bursts containing different number of spikes are not separable, burst duration does not encode information about the tested feature. For a quantitative assessment, we estimated the mutual information between *n* and stimulus feature *F* (see Section Materials and Methods). The results are displayed in Figures 5A7,B7.

Among all the tested stimulus features, the stimulus' phase at burst onset was the one with highest mutual information (0.10 and 0.09 bits/event for MC and IFB models, respectively). Samengo and Montemurro (2010) observed a similar coding scheme between the bursts of pyramidal cell models and the phase of the local field potential. The stimulus amplitude at burst onset has also been suggested as a candidate encoded feature in bust duration (Alitto et al., 2005), but in our model, amplitude seems to be only weakly represented. In turn, Kepecs et al. (2002) reported the slope at burst onset as an informative feature in pyramidal bursting neurons. However, in the case of *I*_*T*_-mediated bursting neurons tested here, slope coding was negligible. Bursts from both models could discriminate *N*_*int*_, that is, the amount of negative charge entering the neuron in the 250 ms prior to burst onset. Interestingly, the encoding of *N*_*int*_ was a time-demanding process, since in both models bursts were found to encode almost no information about the instantaneous amplitude Min of the minimum negative stimulus inside the same window. Finally, the positive charge entering both models was only weakly discriminated by bursts of different spike count.

**Figure 6:**
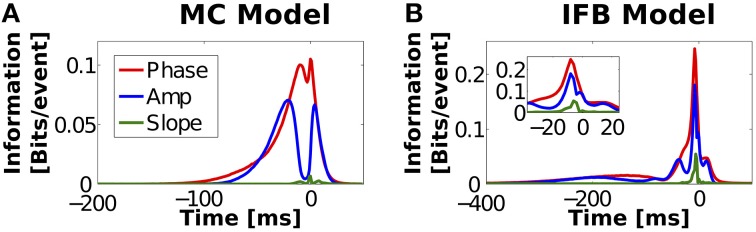
**Information encoded by several instantaneous stimulus features, registered at varying time with respect to burst onset, for the MC (A) and IFB (B) models**. Information transmitted by the discrimination of stimulus amplitude (blue), phase (red) and slope (green) at varying times, for different *n*-values. Digitization *M* = 32, information estimates are shuffle-corrected (see Section Materials and Methods).

The values of the stimulus amplitude, slope, and phase used to calculate the mutual informations reported in Figures 5A7,B7 were calculated at the time of burst onset. However, nothing forbids the size of a given burst to encode the value of a stimulus feature at some other time. The analysis was therefore repeated as a function of the relative timing between burst onset and stimulus feature. The results are shown in Figure 6 with panels A and B corresponding to the MC and IFB models, respectively. We observe an overall tendency of the information to decay for times away from onset. The maximum information, however, is not exactly burst onset but, depending on the feature, it can be slightly before or after. The local maxima for encoded information about stimulus amplitude by MC bursts occur at −21 ms before and 4 ms after onset while IFB bursts produced peak information at −202, −80, −40, −8 before, and 15 ms after burst onset (see blue curves in Figures 6A,B). Interestingly, the information values for amplitude and phase decay over a time range that is significantly longer than the stimulus correlation time or the passive membrane time constant. The only other dynamical candidates for keeping track of the stimulus amplitude for prolonged times are the slow bursting currents. In the MC model, two slow currents exist: *I*_*T*_ and *I*_*Sag*_. The *I*_*Sag*_ current slowly rectifies prolonged (lasting >400 ms) hyperpolarizing membrane voltage deflections and has little effect on the timescales of burst stimulus selectivity. The *I*_*T*_ current has a strong influence on burst stimulus preference. The timescale of this current is controlled by a voltage-dependent variable τ_*h*_ which has an average value of 42 ± 18 ms. For the IFB model, the timescale of the *I*_*T*_ current is given as τ^+^_*h*_ = 100 ms. The timescales of bursting currents therefore closely match the timescales of amplitude discrimination in the IFB and MC models.

For the MC model, amplitude is best encoded in two time regions; −20 ms before and 4 ms after burst onset. Between these peaks lies a trough representing poor discrimination (−10 to 2 ms). It is useful to compare these features with *n*-ETAs in Figure 3A1. The times of greatest information match the regions of *n*-ETAs that show the greatest *n*-dependent shape. Similarly, the area of poor discrimination is due to a *n*-independent depolarizing burst trigger, which can be seen in Figure 3 as the region where all curves overlap. In the case of the IFB model, the information displays multiple peaks of good discrimination (Figure 6B), which also coincide with *n*-dependent areas of *n*-ETAs (Figure 3B1). In both models, phase discrimination shows a similar time evolution as amplitude but with less pronounced blind periods. An instantaneous phase value contains information of past and future stimulus evolution. Consequently phase information presents a smoother profile with a less pronounced minimum. For both models, slope provides only a single small peak of information immediately prior to firing. Slope encoding is connected to phase encoding for slow stimulus components (Samengo and Montemurro, 2010). However, the code fails when the stimulus contains fast Fourier components, as observed here. In Supplementary Figure 6 we show that the results are qualitatively similar when the analysis of instantaneous features is done using input signals with half and double the correlation time used in Figure 5.

### 3.4. Coding time-dependent stimulus features

In Figure 5, the mutual information between *n* and different instantaneous stimulus features was measured. The features were chosen due to their simplicity and their biological relevance, and it is important to notice that they are not independent from one another. For instance, the amplitude at a given time depends on previous values of amplitude and slope, while the phase at a given time depends on the amplitude at previous times. Thus, the distributions in Figure 5 contain a degree of redundancy. None of these features needs to be the precise stimulus property encoded by the bursting neuron. Indeed, the fact that the best encoded instantaneous features are the phase and the negative integral—both quantities defined in terms of the evolution of the stimulus in an extended time window—suggests that the optimally encoded features may well be better described as time-dependent quantities. Moreover, the ETA and the eigenvectors of ETC exhibit structures that span at least 100 ms before burst onset and 50 ms after. The code, hence, most likely involves the evolution of stimulus features, and not just a single feature at a single time.

While it is difficult to treat this problem in full generality, we can still gain insight by restricting our analysis to the family of linear functions of the stimulus. This is equivalent to asking what is the direction in which the stimulus should be projected in order for the projections to generate clusters of minimal overlap (maximally discriminable) when distinguished by the number of spikes *n* of the bursts they trigger. This problem is solved by multi-discriminate analysis (MDA) (Duda et al., 2001; Kepecs and Lisman, 2003).

In brief, a stimulus feature containing *k* time points is represented by a single point in a *k*-dimensional space. Stimuli evoking bursts of *n* spikes should share some common structure and cluster together in this space. The MDA method finds linear axes in *k*-dimension space that best separate different *n*-triggering stimulus clusters. The obtained axes are ranked, with the first axis **V**_1_ pointing along the direction of maximum discrimination, followed by **V**_2_ along the second optimal direction, and so on (Duda et al., 2001). Thus, the first few MDA axes provide the optimal lower-dimensional subspace where to project the stimuli in order to maximally preserve the features that best distinguish the different subsets. We restricted our analysis to the first two MDA axes since *n*-burst triggering stimuli could not be discriminated using the subsequent MDA axes.

It should be noted that the axes obtained from MDA do not need to coincide with the axes obtained from ETC. The latter correspond to directions in stimulus space that trigger bursts of a given duration, no matter whether those same directions also trigger bursts of different durations. MDA is instead concerned with discrimination (as opposed to detection) so only the stimulus directions where different *n*-bursts encode different features are selected.

**Figure 7:**
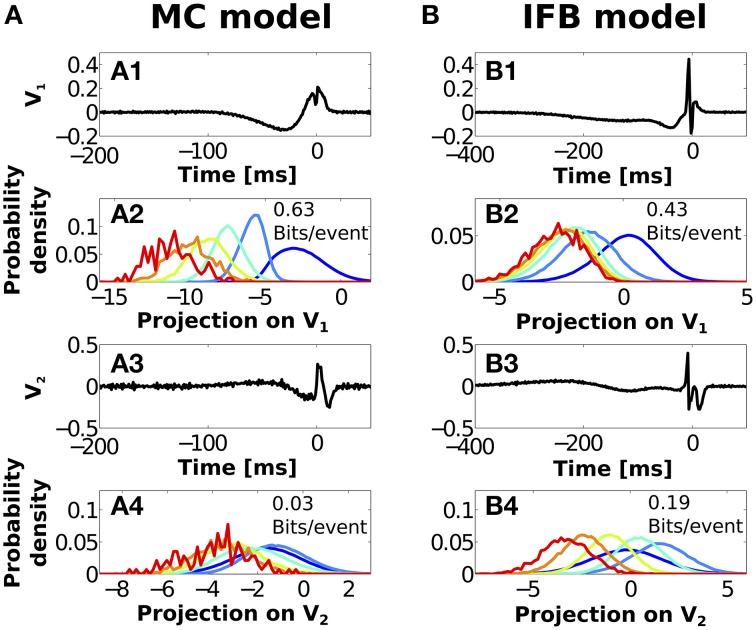
**Optimal stimulus dimensions obtained with multi-discriminant analysis in MC (A) and IFB (B) models. (A1,B1)** Optimal discriminant vector **V**_1_ **(A1,B1)** for each model. **(A2,B2)** Distribution of projections of the stimuli triggering *n*-spike bursts onto **V**_1_. Inset: Mutual information between *n* and projected stimuli. Information was estimated with distributions discretized into *M* = 32 equally populated bins. **(A3,B3,A4,B4)** Same as **A1,B1,A2,B2** but for the second discriminant vector **V**_2_.

Figures 7A1,A3 show the first and second MDA linear vectors (**V**_1_ and **V**_2_) estimated for MC model bursts. Figures 7B1,B3 show the two same vectors for the IFB model. MDA linear vectors can be thought of as a series of stimulus weights across time. The greater the weight at a given time (in absolute value), the stronger the contribution of the stimulus at that time to the discrimination ability of *n*-spike bursts.

For the MC model, the first MDA vector (Figure 7A1) includes non-zero weights spanning −80 ms before burst onset to 15 ms after onset, with a single zero weight at −12 ms. The negative portion of these weights (−80 to −12 ms) matches the time interval where the first ETC eigenvector with *n* = 1 differs from the first eigenvector of all other bursts (Figure 4A1). In this segment, discrimination is likely to operate by distinguishing the stimuli triggering 1-spike bursts from those triggering all other bursts. The positive portion of the weights is associated to the *n*-dependence of the second ETC eigenvector, where the stimulus features terminating bursts vary greatly with *n* (Figure 4A2). Here, discrimination is a graded process, involving all *n*-values. In Figure 7A2 we display the distributions of stimulus projections along the first MDA vector. The curves associated with different *n*-values are clearly more separate than the distributions previously calculated for instantaneous features (compare with Figure 5). The resulting mutual information between *n* and different stimulus projections (0.63 bits/event) far outperforms the discrimination of instantaneous features. We also verified that these results are qualitatively unchanged when using input signals with half or double the correlation time used in Figure 7 (see Supplementary Figure 7).

The second MDA vector of the MC model (Figure 7A3) shows several peaks over a shorter time span. Projection onto the second MDA vector produced largely overlapped distributions (Figure 7A4) with only 0.03 bits/event, implying that the observed structures are not significantly informative. The higher order MDA axes, therefore, only give negligible contributions.

For the IFB model, both the first **V**_1_ and second **V**_2_ MDA vectors (Figures 7B1,B3) are non-zero over a large time span. The vector **V**_1_ begins to depart from zero at −300 ms before burst onset, and continues with a slow trend up to −22 ms. At this time, a sharp peak and trough appear just prior to onset. These temporal scales match the regions where the first ETC vector (Figure 4B1) shows a discrepancy between *n* = 1 and all other *n*-values. Accordingly, the distributions of stimulus projections onto **V**_1_ (Figure 7B2) are separated into two groups: those associated with single spikes (*n* = 1), and those of bursts containing multiple spikes (*n*>1). Once again, the resulting information (0.43 bits/event) markedly outperforms the discrimination of instantaneous stimulus parameters. The second MDA vector **V**_2_ (B3) shows non-zero structure spanning from −250 to 30 ms after onset. This interval coincides with the second ETC eigenvector where *n*-dependent structure is apparent (Figure 4B2). In Figure 7B4, the distribution of stimulus projections onto **V**_2_ are still noticeable separate, producing 0.19 bits/event.

The increased information values obtained from MDA implies that *n*-spike bursts are not specifically tuned to discriminate instantaneous stimulus parameters. The fact that they carry some information about instantaneous features is only a consequence of the way they encode other, more complex, time-dependent features that include the instantaneous ones. Stimulus discrimination by the MC model can be reduced onto a single linear vector whereas the simplified IFB model requires two vectors (B1 and B3).

**Figure 8:**
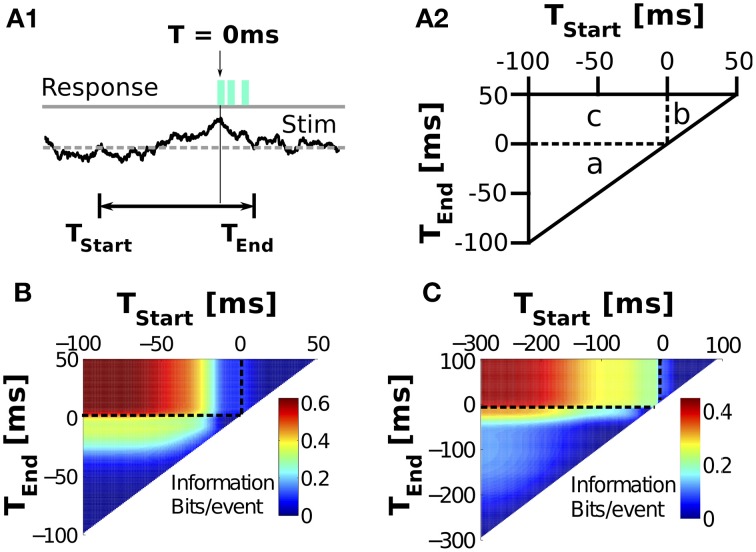
**Dependence of the encoded information on the location of the window used to define the stimulus**. **(A)** Explanatory diagrams illustrating the definition of the windows **(A1)**, and the subdivision of maps **(A2)** into regions: Stimuli may be taken entirely before (**A2** a), after (**A2** b), or astride (**A2** c) burst onset (*T* = 0). Vertical dashed black lines mark *T*_*start*_ = 0 ms and horizontal lines represent *T*_*end*_ = 0 ms. **(B, C)** Information between *n* and **V**_1_-projected stimuli is color-plotted as a function of the location of the times *T*_*start*_ and *T*_*end*_ of the window, for the MC **(B)** and the IFB models **(C)**. Models were driven with stimuli with σ_*OU*_ = 1μA, τ_*OU*_ = 5 ms, μ_*OU*_ = 0μA. Information is calculated with digitization of *M* = 32 bins and shuffle-corrected to account for bias.

The directions obtained with MDA, and consequently, the information values derived from projecting the stimulus on the first MDA vector, depend on the location of the time window used to represent the stimuli. In order to determine the optimal window, we now repeat the analysis, systematically sweeping the location of the first and last times defining the window, *T*_**start**_ and *T*_**end**_. For each window, we project the stimulus on the obtained optimal axis, and calculate the mutual information, as shown in Figure 7A1. The maps are displayed in half planes because by definition, *T*_**start**_ < *T*_**end**_. Figure 8A2 shows a diagrammatic representation, showing that all possible windows can be divided into 3 regions. Windows in region (a) are entirely located before burst onset, since even *T*_**end**_ < 0. Windows in region (b) are entirely located after burst onset, since even *T*_**start**_ > 0. Windows in region (c) are astride burst onset, since *T*_**start**_ < 0 and *T*_**end**_ > 0.

Information maps for the MC and IFB models are plotted in panels B and C, respectively. The information is calculated after projecting all *n*-spike triggering stimuli onto the first **V**_1_ discriminant vector obtained from MDA. Information maps for the **V**_2_ vector are given as supplemental information. The data processing inequality (Cover and Thomas, 2006) ensures that shorter time windows cannot encode more information than longer ones. Therefore, the greatest information occurs for the largest stimulus windows, that is, when *T*_**start**_ is minimal and *T*_**end**_ is maximal (far top left of Figures 8B,C). As *T*_**end**_ decreases and approaches *T*_**start**_ (with *T*_**start**_ kept constant), the window shrinks and the information necessarily falls down to zero. The same happens when *T*_**start**_ is increased and approaches *T*_**end**_. The optimal window is the one encompassing all informative events, and no uninformative events. For the MC model, the optimal windows are around *T*_**start**_ ≈ −70 ms, and *T*_**end**_ ≈ 20 ms. For the IFB model, the corresponding values are −200 and 30 ms. In both models, *n*-burst events are more sensitive to the stimulus preceding burst onset than those coming after.

In the supplementary information we analyze the information maps for the tonic firing MC-T (A) and IFB-T (B) models. Removing the burst currents diminished the number of generated bursts, but did not eliminate them completely: Tonic firing may occasionally still produce packets of high-frequency discharges. The amount of information encoded in the remaining tonic bursts, however, dropped drastically. Interestingly, tonic bursts lost the ability to discriminate pre-onset stimulus features, but retained the post-onset features. Hence, the information encoded by time windows located in different regions of the maps of Figure 8 may be underpinned by different dynamic mechanisms.

## 4. Discussion

In this paper, we extended previous studies of thalamic burst coding (Reinagel et al., 1999; Weyand et al., 2001; Oswald et al., 2004; Alitto et al., 2005; Marlinski and Beloozerova, 2014) by showing that thalamic bursts produced by two neuron models (Wang, 1994; Smith et al., 2000) form a graded, *n*-spike burst code. We report that these bursts reliably modify their internal spike count (*n*) in response to different stimulus deflections that span up to 300 ms prior to burst onset. Moreover, we show that this code can induce stimulus-modulated correlations that are similar to the ones found in *in-vivo* thalamic neuron responses (Montemurro et al., 2007a). Interestingly, the code can be simulated using a highly simplified thalamic relay model (Smith et al., 2000).

We drove a simplified integrate-and-fire or burst (IFB) and a more complex multi-conductance (MC) model with stochastic stimuli. The two models produced responses containing significant amounts of information through stimulus-modulated spike correlations (Δ*I*), which existed only when bursts were present in the response. Work by Montemurro et al. (2007a) found that Δ*I* accounted for 6.6% of all information transmission in *in-vivo* thalamic neurons. Here we suggest that this information is carried by bursts. To reveal the meaning of the encoded information, we analyzed the stimuli that evoked different *n*-spike bursts. We found that bursts from both models encoded a number of instantaneous stimulus quantities, such as the stimulus amplitude at burst onset (Eyherabide et al., 2008), slope (Kepecs et al., 2002), or phase (Samengo and Montemurro, 2010). However, when *n*-spike bursts were assumed to discriminate temporally structured aspects of the stimulus, the encoded information increased dramatically, by a factor of six. Specifically, we found that the *n*-spike burst code provided information about the behavior of the stimulus both before and after burst onset.

The *n*-spike burst code was sustained by two different dynamic processes. Pre-onset stimulus discrimination was controlled principally by stimuli tuning the slow *I*_*T*_ current. The encoding of post-onset stimuli was not mediated by this current, but rather, by the tonic prolongation of firing. Bursts are therefore not rigid events, instead they are generated by mechanisms that are sensitive to stimulus fluctuations. The extent of this stimulus sensitivity *in-vivo* is not well-understood but here we provide a plausible explanation for previous observations of spike correlation coding (Montemurro et al., 2007a).

### 4.1. Control of burst coding

Our results show that thalamic models require certain stimuli before and after firing in order to produce bursts of a given spike count *n*. In both models, the origin of this behavior lies in the hyperpolarization-sensitive *I*_*T*_ current underpinning bursting (Alexander et al., 2006; Coulon et al., 2009; Tscherter et al., 2011). The *I*_*T*_ current slowly becomes primed by hyperpolarizing input and then, once triggered, depolarizes the membrane sufficiently to fire bursts. In both models, the *I*_*T*_ current is modeled by an activation and inactivation variable. However, these variables are described with different levels of biological realism. For the MC model, the activation variable is a sigmoid function of the membrane voltage, while the inactivation variable is a slow function of membrane voltage governed by a voltage-dependent time constant (Wang, 1994). In the IFB model, the *I*_*T*_ current is highly simplified. The activation variable is a simple Heaviside switching function based on membrane voltage while the inactivation variable activates and decays based on two fixed time constants (Smith et al., 2000). In spite of these differences in biological realism, the two models show remarkably similar stimulus coding. For example, both models transmit similar amounts of information through stimulus-modulated correlations (Figures 2A4,B4); fire *n*-spike bursts that are evoked by similar instantaneous stimulus quantities (Figures 3A2–A6,B2–B7) or time-dependent stimulus features (Figure 4); and employ a *n*-spike burst code that discriminates stimuli before and after burst onset (Figures 7A1,A3,B1,B3). Hence, the computational properties of thalamic neurons are well-captured by a simple model containing an *I*_*T*_ current.

### 4.2. The role of the *n*-spike burst code

In previous studies, thalamic bursts were reported to be informative because they accurately mark the timing of relevant stimuli (Guido and Weyand, 1995; Reinagel et al., 1999; Alitto et al., 2005). Clearly, bursts that also reliably alter their internal spike count can encode extra information, reporting categorical information about the encoded stimulus (Eyherabide et al., 2009; Eyherabide and Samengo, 2010). Since thalamic bursts can fire reliably to synaptic input (Alitto et al., 2005; Bessaïh et al., 2008), we hypothesize that the timing of thalamic bursts marks the onset of a stimulus, whereas the number of spikes *n* contained in the burst encodes other properties of the stimulus spanning multiple times.

By using bursts in this way, thalamic neurons can effectively filter the stimulus, reducing its dimensionality to a few linear axes, and then encoding regions along these axes with an easily decodable set of different *n*-spike bursts. Spike codes can also represent a reduced set of stimulus dimensions (Agüera y Arcas et al., 2001, 2003; Agüera y Arcas and Fairhall, 2003). However, since bursting partly relies on the activity of slow currents, bursts can encode stimulus features that fall outside of the stimulus preference of tonic spikes (Eyherabide et al., 2008). To compare these coding schemes, we tested the stimulus discrimination of tonic firing MC-T and IFB-T models. These models fire groups of high-frequency tonic spikes that resemble bursts but do not show preference for previous membrane hyperpolarizations, they simply fire when driven by strong depolarizing stimuli. In the Supplementary Material, the information maps obtained with different stimulus windows are shown for the MC-T (A) and IFB-T (B) models. Stimulus discrimination only occurs once the stimulus windows contain post-onset stimuli, meaning that burst-like groups of tonic spikes cannot reproduce the pre-onset stimulus discrimination associated with intrinsic bursting.

### 4.3. Other burst codes

Other burst codes are also possible, such as burst duration (Kepecs et al., 2002; Kepecs and Lisman, 2003, 2004); or burst spike density (also known as burst ISI coding) (Oswald et al., 2007; Marsat and Pollack, 2010). In the supplemental information, we test these possibilities by reclassifying bursts using these structural properties. Even when bursts were classified into 20 different symbols (by grouping bursts of different durations or average ISIs into equally spaced bins), these “finer” burst codes did not provide extra information compared with the simpler *n*-spike burst code. Therefore, other aspects of thalamic burst structure do not contribute to a stimulus-discriminating code.

### 4.4. Implications for real neurons

Bursts are known to fulfill a number of functions within the thalamus ranging from non-perceptive tasks such as maintaining large-scale rhythmic activity during sleep (Domich et al., 1986; Steriade et al., 1993; Kiss et al., 1995) to marking the onset times of salient stimuli (Steriade and Llinas, 1988; McCormick and Feeser, 1990). However, the internal structure of thalamic bursts was never considered relevant for encoding stimulus information. The observation of small yet significant amounts of information transmitted through stimulus-modulated spike correlations by *in-vivo* thalamic neurons (Montemurro et al., 2007a) raised the possibility that thalamic burst structure may play an encoding role. We confirmed this possibility by finding that thalamic neuron models transmit information via stimulus-modulated changes in burst spike count. This coding was a consequence of the hyperpolarization-sensitive *I*_*T*_ current and could be replicated in a highly simplified model of thalamic neurons. Therefore, neurons possessing such a current would have the machinery to encode information using the *n*-spike burst code.

A major advantage in carrying stimulus information using this code is that different *n*-spike bursts can be easily decoded by downstream neurons. Work by Swadlow and Gusev (2001) has shown that thalamic bursts can preferentially evoke depressing neocortical synapses. This is due to the extended silence prior to thalamic bursting which elevates synaptic depression. It is possible that larger bursts (encoding deeper or more prolonged periods of hyperpolarization) can activate this type of synapse more efficiently and possibly produce multiple post-synaptic spikes. Facilitating synapses require a succession of pre-synaptic spikes before they fire. This affectively blocks the transmission of single spikes but allows post-synaptic firing to bursts (Lisman, 1997). It stands to reason that larger *n*-spike bursts will evoke post-synaptic spikes through either type of synapse and transmit the information carried in the *n*-spike code.

## 5. Conclusions

Our results show that neurons containing *I*_*T*_ currents can use their burst spike-count to encode stimulus features not encoded in tonic spikes alone. This coding explains the origin of the information encoded in stimulus-dependent spike correlations that have been observed in thalamus (Montemurro et al., 2007a). The stimulus preference of different *n*-spike bursts changed in a graded way. This allowed *n*-spike bursts to encode a number of instantaneous stimulus properties including the amount of hyperpolarizing charge entering the neuron prior to onset, the stimulus amplitude and the stimulus phase at burst onset. However, no single stimulus quantity was strongly discriminated by *n*-spike bursts (discrimination of phase offered ≈0.1 Bits/event). Therefore, assuming bursts encode instantaneous stimulus properties may underestimate their capacity to encode stimuli. In support of this idea, we found that *n*-spike bursts discriminated instantaneous stimulus features (particularly amplitude and phase) over a whole range of times. This suggests that burst size is tuned by multiple stimulus factors that cannot be quantified by a single 1-dimension feature. To analyse stimulus discrimination without this limitation, we used MDA to reduce the stimulus dimensionality into linear axes (discriminant vectors) along which the stimulus distributions evoking different *n*-spike bursts were most separable (Duda et al., 2001).

Along each of these axes, the encoded information was found to be approximately 6 times greater than the one obtained for instantaneous stimulus parameters, for both models and for the specific stimulus parameters used in the main analysis. Therefore, stimulus encoding is badly underestimated by relating *n* to simple stimulus features.

Stimuli occurring before and after burst onset were discriminated by *n*-spike bursts. Whereas tonic models only discriminated stimuli following the onset of firing. Therefore, pre-onset stimulus discrimination occurs because prior to burst onset, the stimulus may modulate the amplitude or the duration of the *I*_*T*_ current. Post-onset stimulus discrimination results from the stimulus directly cutting/prolonging spiking after burst onset. This dual stimulus dependence does not rely on the complexity of the model, since both MC and IFB systems display similar results, albeit at different timescales. Overall, our results support the hypothesis that the key computational features of bursting thalamic neurons are underpinned by the dynamics of the *I*_*T*_ current, and are only marginally dependent on other biological details.

## Author contributions

Conceived and designed the experiments: DE, MM. Performed and analyzed experiments: DE. Contributed to the manuscript figures and text: DE, IS, MM. Wrote the paper: DE, IS, MM. Proofread the manuscript: DE, IS, MM.

## Funding

This work was supported by the Biotechnology and Biological Sciences Research Council (BBSRC), UK. Grant code: BB/D526561/1. We also thank the Agencia Nacional de Promoción Científica y Tecnológica and the Ministerio de Ciencia y Tecnología of Argentina.

### Conflict of interest statement

The authors declare that the research was conducted in the absence of any commercial or financial relationships that could be construed as a potential conflict of interest.
