# Supplementary material for: Thalamic neuron models encode stimulus information by burst-size modulation
Source: Front Comput Neurosci. 2015 Sep 23;9:113. doi: 10.3389/fncom.2015.00113 (PMC4585143; doi:10.3389/fncom.2015.00113)
Supplement: Supplementary file 1 [file DataSheet1.PDF]

frontiers

Supplementary Material: Thalamic neurons encode stimulus information by burst-size modulation

Daniel H. Elijah [1,*], Inés Samengo [2] and Marcelo A. Montemurro [1]

[1] *The University of Manchester, Faculty of Life Sciences, Manchester, United Kingdom.*
[2] *Instituto Balseiro and Centro Atómico Bariloche, San Carlos de Bariloche, Río Negro, Argentina.*

Correspondence*:
Daniel H. Elijah
The University of Manchester, Faculty of Life Sciences, Manchester, United Kingdom., daniel.elijah@manchester.ac.uk

SUPPLEMENTARY DATA

FEATURE DIGITIZATION AND BIAS

Bias is a major problem when estimating information from a limited number of samples. It is a systematic error that produces overestimated information values (Panzeri et al., 2007). The amount of bias depends on the sample size (a factor that may be difficult to alter in an experimental situation) and the digitization resolution M. The variable F must be digitized into M bins to give $\tilde{F}$ from which we obtain the probability distributions $P(f)$ and $P(f \mid n)$. If M is set at a large value compared with the sample size, then the entropy $H(\tilde{F} \mid N)$ is typically under-estimated to a greater degree than $H(\tilde{F})$, meaning $I(\tilde{F}; N)$ is overestimated. If M is too small then $I(\tilde{F}; N)$ underestimates the true value of information. M must therefore be set to an appropriate value to provide an accurate information estimate without significant bias. In Supplementary Figure 1, we plot the information estimates for a range of M using both MC (solid) and IFB (dashed) models. To account for the effects of estimation bias, we subtract a shuffled information estimate that contains only bias from the original information estimate. The original information estimates are shown with black lines, grey lines represent bias-corrected information estimates.

When $M < 2^3$, both estimations of information underestimate the true values. The estimates get better when $M \to 2^8$. However, when $M > 2^8$, the two estimates diverge, with the non-corrected estimate increasing sharply, while the bias subtracted estimate remains approximately constant. Although it would be feasible to use the shuffled corrected value when $M > 2^8$, computation time quickly becomes problematic. We therefore set $M = 2^5$ throughout this study. This provides a close estimate of the true information, fast computation times, and minimal bias in our calculations. This test was repeated with similar results for the MDA information estimates.

READING THE THALAMIC BURST CODE

The internal structure of bursts can be read using a number of different codes. Of these, the burst spike count code is the simplest and arguably the easiest to decode. However, distinguishing bursts by their spike count only allows a limited number of distinct bursts to become available for stimulus encoding.

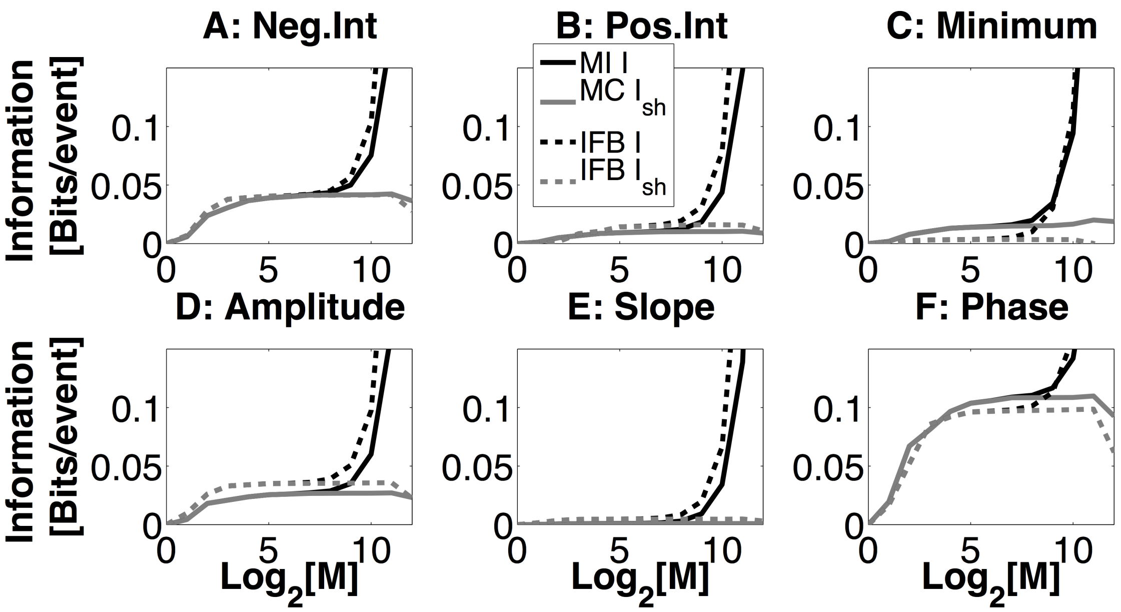

Supplementary Figure 1. Estimates of information available through the discrimination of 1-dimensional stimulus features as a function of digitization resolution M. Panels represent the information measured for different 1-dimensional stimulus features, data for the MC and IFB models are shown as solid and dashed lines respectively. Black curves represent uncorrected estimates of information, gray curves show shuffle-corrected estimates. (A) negative stimulus charge prior to onset; (B) positive stimulus charge following onset; (C) stimulus minimum prior to onset; (D) stimulus amplitude; (E) stimulus slope; (F) phase. (D-F) are calculated at burst onset.

This problem is bypassed if bursts are distinguished using an aspect of their structure that can vary continuously. Examples of such continuous aspects include burst duration (Kepecs et al., 2002; DeBusk et al., 1997) and intra-burst spike density codes (or ISI codes) (Oswald et al., 2007).

These are potentially more informative than the ‘discrete’ burst-spike count code, because the number of different possible responses increases exponentially with spike timing resolution. For duration or burst ISI codes to outperform the n-spike burst code, these continuous features of bursts must be tightly tuned to the stimulus compared with bursts classified by their spike count.

We test the viability of burst duration and mean ISI codes by discretizing them with two different resolutions. We digitized across the range of burst durations $(0 - 40\,\mathrm{ms})$ and average ISIs ($0\,\mathrm{ms}$ (for single spikes) to $10\,\mathrm{ms}$). The coarsest resolution splits all burst durations and mean ISIs into 6 bins; corresponding to the maximum number of symbols produced by burst spike count n. The second resolution contains 20 bins, allowing any fine stimulus discrimination to become apparent. Course binning of bursts using their duration provided 0.47 and 0.23 bits/event for the MC and IFB models, the same binning for the ISI code provides 0.38 and 0.29 bits/event. Finer binning of the duration code provides 0.55 and 0.59 bits/event whilst the ISI code only provides 0.51 and 0.58 bits/event.

Therefore, reading bursts using their duration or spike density produced no more information compared with the n-spike burst code (producing 0.66 and 0.62 bits/event for the MC and IFB models). The n-spike code therefore allows optimum transmission of stimulus information through a limited set of burst symbols.

OPTIMAL DISCRIMINATION PRODUCED BY THE SECOND MDA DISCRIMINANT VECTOR (V_2)

In Figure 8 (B and C), the information available by projecting n-triggering stimuli onto the first discriminant axis $(\mathbf{V}_1)$ was shown. However in Figure 7 (A4 and B4), n-burst triggering stimuli are also discriminated along the second MDA discriminant axis $(\mathbf{V}_2)$, albeit to a lesser degree.

In Supplementary Figure 2, the information computed by projecting along the $\mathbf{V}_2$ vector was explored further for both models using information maps.

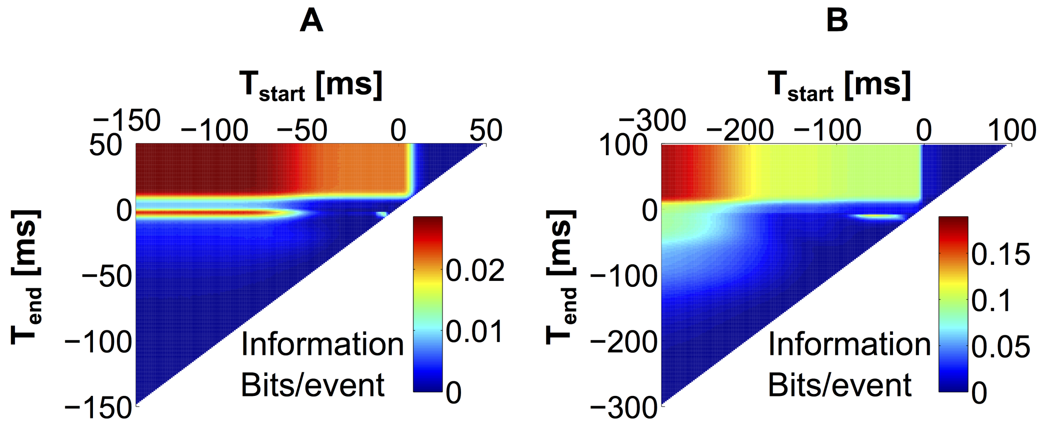

Supplementary Figure 2. Information computed by projecting the stimuli along the second MDA eigenvector V_2, for different locations of the temporal window. Maps are separated into regions as in Figure 8 (A2). Information estimates are calculated with digitization $M = 32$ bins and are shuffle corrected.

Information maps produced by $\mathbf{V}_2$ display a different structure from those generated with the $\mathbf{V}_1$ vector. For the MC model, $\mathbf{V}_2$ maps display no informative stimulus regions before -5ms, but do display a brief informative region between -5ms and 0ms. This is related to the brief negative deflection in the MDA $\mathbf{V}_2$ vector (shown in Figure 7 (A3)) at these times. Information increases predominantly once the stimulus window includes post-onset stimuli. In Figure 7 (A4), $\mathbf{V}_2$ projections of n-triggering stimuli separate when $n > 2$. These maps demonstrate that along this axis, stimuli prolong MC burst firing following onset.

IFB model $\mathbf{V}_2$ maps also show significantly different structure compared with their $\mathbf{V}_1$ counterpart (compare with Figure 8 (C) and Supplementary Figure 2 (B)). Informative stimulus regions that extended to -200ms for $\mathbf{V}_1$ maps now extend up to -100ms and contain proportionally more information compared to the largest stimulus window (given in the top left of the map). This proportionally stronger discrimination of past stimuli further supports the result that burst size is modulated by the stimulus via the I_T current. However, complete tuning of burst size can only be achieved when both pre- and post-onset stimuli are accounted for, meaning that the combination of I_T current modulation and direct stimulus drive following onset are required for tuning burst size.

DISCRIMINATION OF STIMULI BY TONIC MODELS

Supplementary Figure 3 shows stimulus discriminant information maps for tonic-firing MC-T (A) and IFB-T (B) models. Maps were calculated using the $\mathbf{V}_1$ discriminant vector, stimulus projections onto the remaining vectors produced no stimulus discrimination. These models cannot fire I_T-mediated (intrinsic)

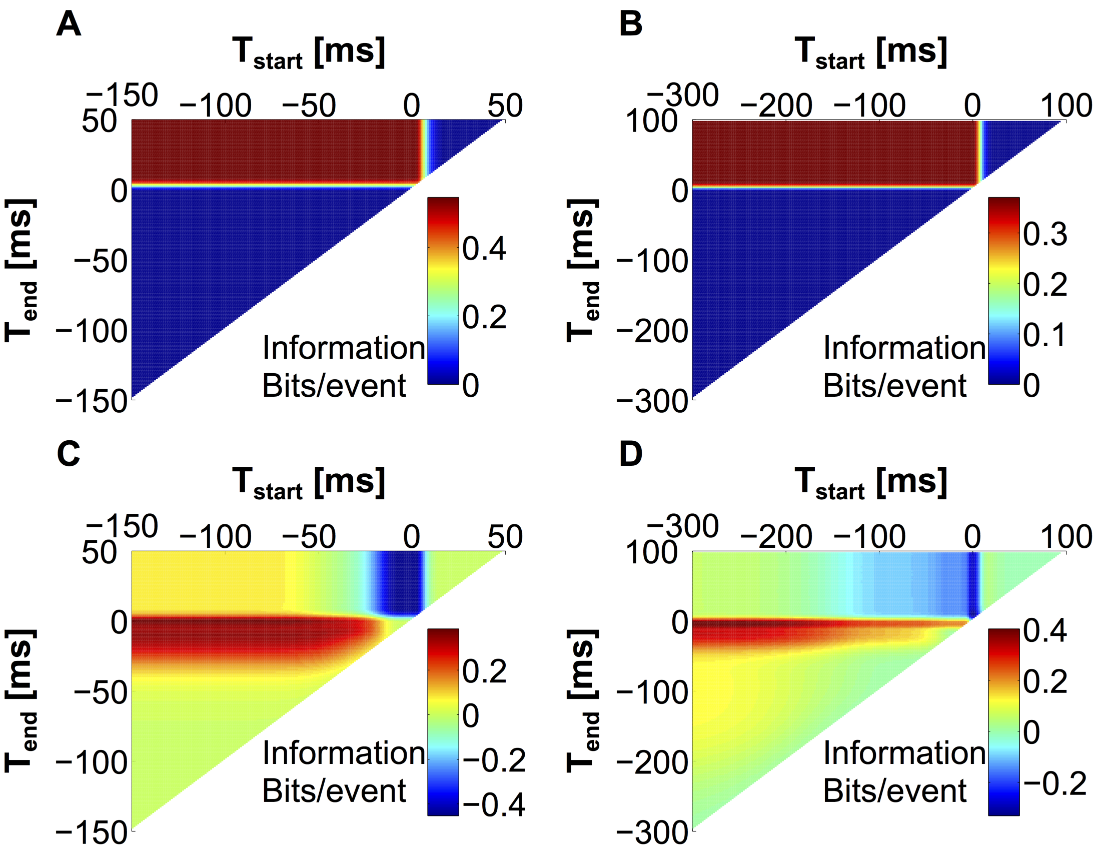

Supplementary Figure 3. MDA information maps plotted for the tonic MC-T (A) and IFB-T (B) models. Tonic information maps (A and B) are subtracted from their bursting counterparts (Figure 8 (B and C)) to produce information-subtraction maps for the MC (C) and IFB (D) models. Informative stimulus discrimination is marked by non-zero information values. Information estimates are shuffle corrected. Digitization resolution is $M = 32$ bins.

bursts but instead fire tonic spikes at high rate. These high rate tonic 'bursts' are defined using the same 10ms ISI threshold used for intrinsically bursting models. Discrimination maps for both tonic models show informative stimulus windows only at times immediately following burst onset (typically after 10 ms). Stimulus windows covering pre-onset stimuli remained undiscriminated by tonic responses. To highlight differences between the stimulus discrimination of bursting and tonic models, we subtracted their information maps. Subtracted maps are displayed in Supplementary Figure 3 for the MC (C) and IFB (D) models. Positive areas indicate stimulus regions modulating intrinsic bursts, negative areas show stimulus regions modulating tonic bursts. Subtracted maps can be split into two regions depending on the dynamic mechanism underpinning stimulus preference. Stimuli preceding bursting are discriminated by intrinsic bursts produced by models containing an I_T current. Stimuli occurring after burst onset alter n by forcing or inhibiting burst firing without influencing bursting currents directly. This occurs in both intrinsic and tonic bursts but this form of stimulus modulation is more pronounced for tonic bursts. Negative regions in panels (C) and (D) at $T_{end} > 0$ ms extend prior to burst onset ($T_{Start} < 0$ ms) because intrinsic bursts exhibit a blind region at these times; formed by stimuli acting only to dis-inhibit bursting currents. Tonic model responses are more informative in these regions because stimulus windows encompass burst firing.

CONTRIBUTION OF n-SPIKE BURSTS TO STIMULUS DISCRIMINATION

The n-spike burst code is informative because its individual coding symbols (represented by different n-spike bursts) occur with non-zero probabilities and are locked to different stimulus distributions. In this section, we briefly analyze the contribution each n-spike burst has to the n-spike code from these two perspectives.

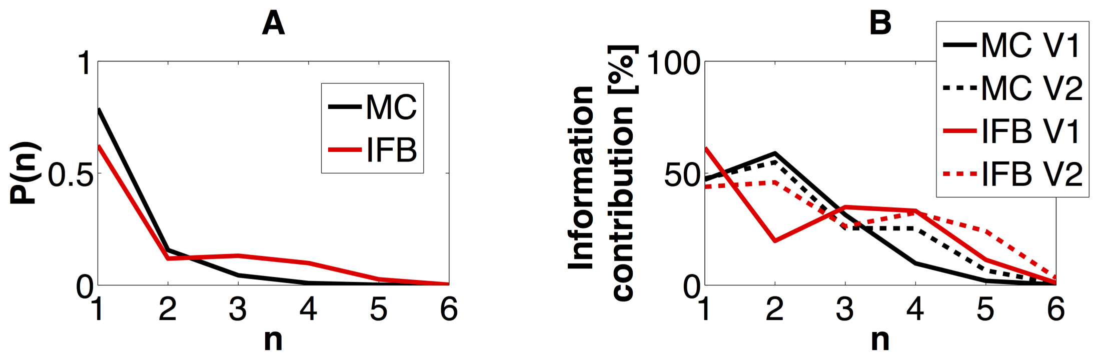

Supplementary Figure 4. Probability of observing n-spike bursts (A) and their contribution to stimulus discrimination (B). The contributions of different n-spike bursts are shown in terms of firing probability (A) and the information lost if n is replaced by a shuffled selection of all $n \in N$ (B). In (B), information estimates are shuffle-corrected, digitization resolution is $M = 32$ bins.

In Supplementary Figure 4 (A), the probability of observing different n-spike bursts is shown for MC (black curve) and IFB (red curve) models. An efficient code will use symbols with an equal probability of occurrence while a non-informative code will employ one symbol and neglect all others. In Supplementary Figure 4, single spikes from MC and IFB models are the most common symbol (with $P(n = 1) = 0.79$ (MC) and $P(n = 1) = 0.62$ (IFB)). $P(n)$ then decreases with increasing n. Therefore, the amount of decodable information carried by the n-spike burst code is restricted by the rarity of large bursts. The information carried by a given n-spike burst also depends on how well separated its evoking stimulus distribution is from the stimulus distribution evoking all bursts.

In Supplementary Figure 4 (B), the amount of information contributed by individual n-spike bursts to the n-spike burst code is found by replacing a given n with a randomized selection of $n \in N$. As a result, the stimuli previously associated with n cannot be discriminated, reducing the amount of information carried by the n-spike burst code. In (B), this lost information is expressed as a percentage of the information gained through MDA linear discrimination. Smaller events $n < 3$ contribute more information for both MC (black curve) and IFB (red curve) models. This is true when stimuli are projected onto the first (solid line) or second (dashed line) MDA discriminant vectors. Interestingly, 2-4 spike bursts only represent a small fraction of the total number of observed responses (see Supplementary Figure 4 (A)), yet they contribute a disproportionally large amount of information (Supplementary Figure 4 (B)). This is evident by the relatively modest drop in information with increasing n in (B) compared with the firing probability (A). The n-burst code therefore relies on the entire range of n-spike bursts and the information it transmits will be underestimated if larger bursts are ignored.

SINGLE SPIKE EVENTS

In this paper, single spike events were defined as being separated by more than 10ms from previous or following spikes. Using this definition, single spikes form a single coding symbol. However, n-spike

events can also be distinguished using the strength of underlying I_T current activity at n-spike burst onset. If this and ISI threshold sorting are used together then single spikes can be separated into two event types. The first is a tonic spike; a single spike fired without the influence of bursting currents. The second is a 1-spike burst. This event contains a single spike fired during I_T current activation. It is typically proceeded by a stimulus hyperpolarization before being triggered by a sharp depolarization similar to small bursts. Following the firing of the first spike, the stimulus hyperpolarizes the membrane, preventing further firing. These bursts shared stimulus preferences with 2-spike bursts rather than tonic spikes. In Supplementary Figure 5, the ETAs of these dynamically different events have been plotted for the MC (A) and IFB (B) models.

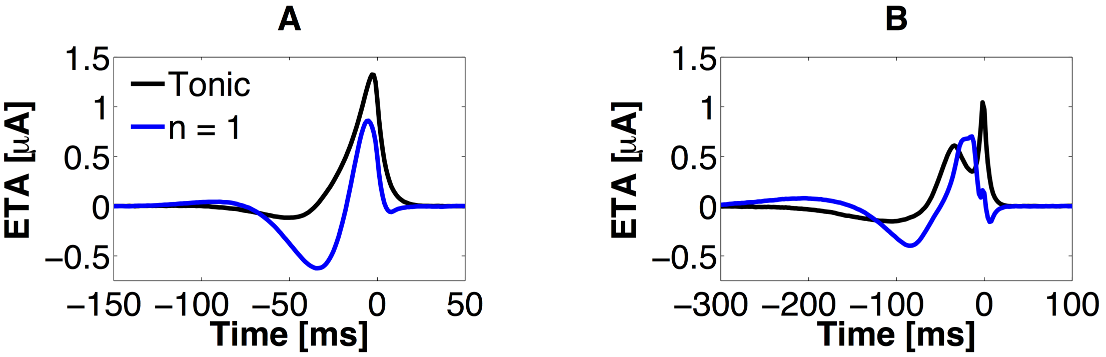

Supplementary Figure 5. **ETAs of single spike events generated by the MC (A) and IFB (B) models.** Black curves show ETAs of tonic spikes $(n = 1)$, defined as single spike events that are both separated from other spikes by $\geq$ 10ms and fired when $I_T < 3\mu A$. Blue curves show ETAs of 1-spike bursts, defined as single spike events fired when $I_T \geq 3\mu A$.

In this paper, we combined these events into a single spike coding symbol on the basis that downstream neurons distinguish different neuron responses using only n. However, downstream neurons may distinguish tonic spikes from 1-spike bursts if they account for previous spiking activity. In Supplementary Figure 5, 1-spike bursts were preceded by deeper stimulus hyperpolarizations, this may indicate longer ISIs preceding 1-spike bursts compared to tonic spikes. This difference may be detectable for downstream neurons. Despite this possibility, it is not known if 1-spike bursts actually exist in-vivo or whether a stimulus coding role exists for them.

ROBUSTNESS OF INFORMATION ESTIMATES

Throughout this paper, we have simulated the synaptic drive of neurons using an Ornstein-Uhlenbeck (OU) stochastic process. A key aspect of the OU process is the timescale of stimulus correlation which was set at $\tau_{OU} = 5$ ms to best replicate the millisecond time-scale of synaptic input fluctuations. However, the time-scales of these fluctuations may vary depending on factors including the rhythmicity of synaptic input, presence of neural modulators, electrical properties of the neuron, etc. To find how robust our results are to changes in stimulus correlation, we recalculate the stimulus information gained by reading the n-spike burst code while changing the stimulus correlation time constant τ_{OU} to 2.5ms and 10 ms.

In Supplementary Figure 6, we show the information gained about 1-dimensional stimulus features by reading n-spike bursts. Results for the MC (A) and IFB (B) models are shown. The top row corresponds to stimuli with a correlation time of $\tau_{OU} = 2.5$ ms, middle row; $\tau_{OU} = 5$ms (as shown in Fig. 5(A7, B7)), bottom row shows $\tau_{OU} = 10$ ms.

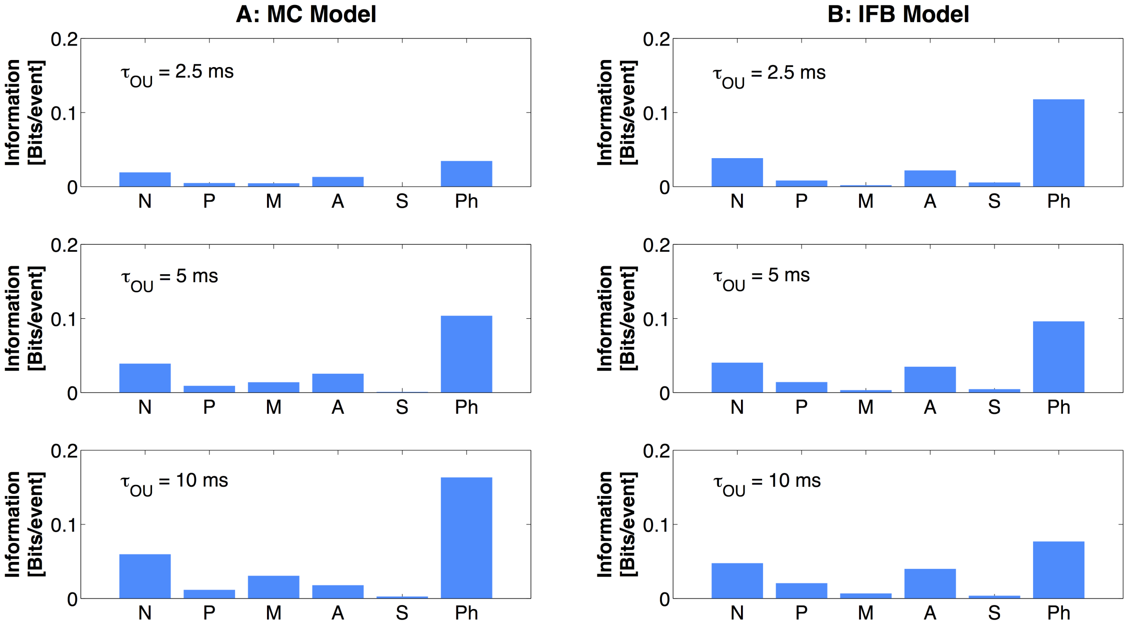

Supplementary Figure 6. Effect of stimulus correlation on the information encoded by the n-spike burst code about 1-dimensional stimulus features. The top row corresponds to stimuli with short ($\tau_{OU} = 2.5$ ms) correlation, middle row $\tau_{OU} = 5$ ms and the bottom row shows results for stimuli with a long ($\tau_{OU} = 10$ ms) correlation timescale. Information was estimated by binning stimulus features using $M = 32$ bins, all estimates were shuffle corrected.

As stimulus correlation is lengthened, the information transmitted by the n-spike code about 1-dimensional stimulus features increases. However, this change is marginal and does not apply to some features, such as stimulus phase driving the IFB model and stimulus amplitude driving the MC model. Increases in information occur for two reasons. Firstly, increasing stimulus correlation increases the dependence between 1-dimensional stimulus features and previous stimulus behaviour. Since the n-spike code is sensitive to stimulus behaviour spanning multiple times, this increase in stimulus correlation increase information. Secondly, increasing stimulus correlation promotes prolonged stimulus deflections which in turn increases the firing rate of larger bursts. Being more numerous, these large bursts now have a contribution to the n-spike burst code, increasing information. The increased dependence on stimulus history may also reduce information if that history does not modulate n, such as stimulus regions prior to burst onset (see Figure 6).

Since correlation affects the stimulus structure over a range of times, it is useful to discuss the effect changing stimulus correlation has on the discrimination of multiple-time stimulus fluctuations. In Supplementary Figure 7, we show the stimulus projections that produce maximal discrimination (using MDA) for stimuli set with $\tau_{OU} = 2.5$ ms (top row), $\tau_{OU} = 5$ ms (middle row) and $\tau_{OU} = 10$ ms (bottom row). Information values are inset within each panel.

As with the 1-dimensional stimulus features, there is only a marginal change in information, which increases with increasing τ_{OU}. The position of each n-spike triggering stimulus projection is conserved for different τ_{OU} meaning that the increase in information is due to increasing numbers of larger bursts.

We can therefore conclude that stimulus correlation has a small, predictable effect on the amount of information contained in the n-spike burst code.

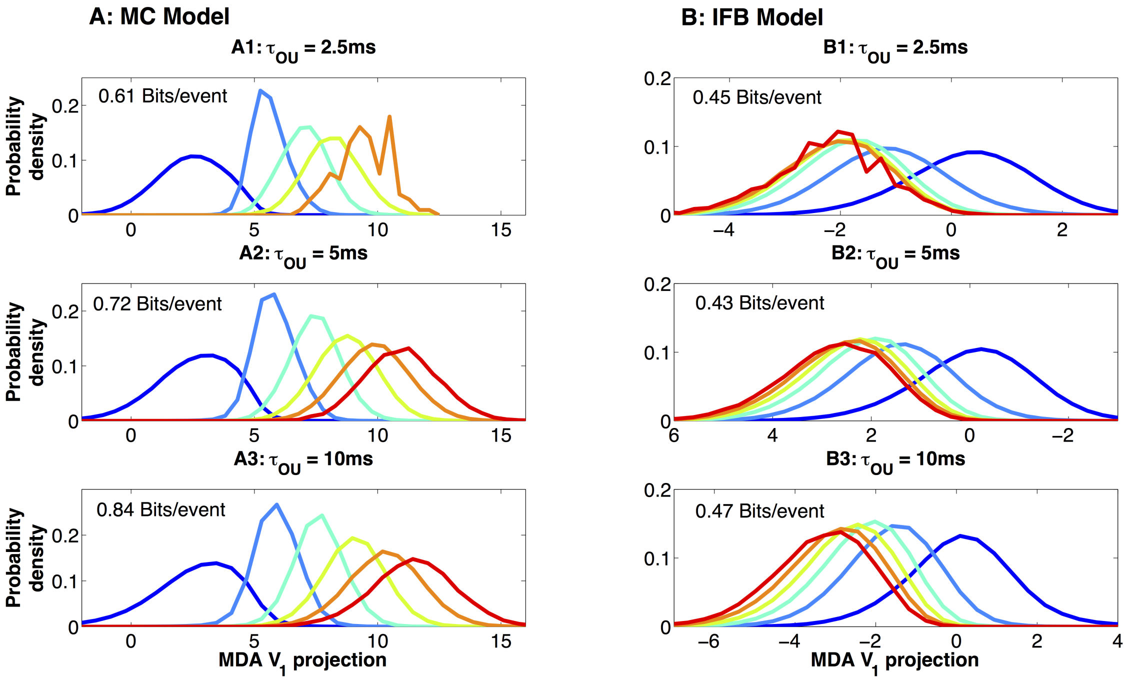

Supplementary Figure 7. **Stimulus projections that result in optimal discrimination calculated for stimuli with different correlation time constants.** Top row: $\tau_{OU} = 2.5$ ms, middle row: $\tau_{OU} = 5$ ms, bottom row: $\tau_{OU} = 10$ ms. Information values are estimated using $M = 32$ bins and are shuffle corrected.

REFERENCES

DeBusk, B. C., DeBruyn, E. J., Snider, R. K., Kabara, J. F., and Bonds, A. B. (1997). Stimulus-dependent modulation of spike burst length in cat striate cortical cells. *J Neurophysiol*, 78:199–213.

Kepecs, A., Wang, X. J., and Lisman, J. (2002). Bursting neurons signal input slope. *J Neurosci*, 22(20):9053–62.

Oswald, A. M., Doiron, B., and Maler, L. (2007). Interval coding. I. burst interspike intervals as indicators of stimulus intensity. *J Neurophysiol*, 97(4):2731–43.

Panzeri, S., Senatore, R., Montemurro, M. A., and Petersen, R. S. (2007). Correcting for the sampling bias problem in spike train information measures. *J Neurophysiol*, 98(3):1064–72.
